# Bimetallic nanosized solids with acid and redox properties for catalytic activation of C–C and C–H bonds†
†Electronic supplementary information (ESI) available: General procedures, additional figures and tables, compound characterization and NMR copies. See DOI: 10.1039/c6sc03335k


**DOI:** 10.1039/c6sc03335k

**Published:** 2016-08-26

**Authors:** Jose R. Cabrero-Antonino, María Tejeda-Serrano, Manuel Quesada, Jose A. Vidal-Moya, Antonio Leyva-Pérez, Avelino Corma

**Affiliations:** a Instituto de Tecnología Química , Universitat Politècnica de València-Consejo Superior de Investigaciones Científicas , Avda. de los Naranjos s/n , 46022 , Valencia , Spain . Email: acorma@itq.upv.es ; Email: anleyva@itq.upv.es ; Fax: +34 9638 77809 ; Tel: +34 9638 77800; b King Fahd University of Petroleum and Minerals , P. O. Box 989 , Dhahran 31261 , Saudi Arabia

## Abstract

A new approach is presented to form self-supported bimetallic nanosized solids with acid and redox catalytic properties. They are water-, air- and H_2_-stable, and are able to activate demanding C–C and C–H reactions. A detailed mechanistic study on the formation of the Ag–Fe bimetallic system shows that a rapid redox-coupled sequence between Ag^+^, O_2_ (air) and Fe^2+^ occurs, giving monodisperse Ag nanoparticles supported by O-bridged diatomic Fe^3+^ triflimides. The system can be expanded to Ag nanoparticles embedded within a matrix of Cu^2+^, Bi^3+^ and Yb^3+^ triflimide.

## Abstract 2

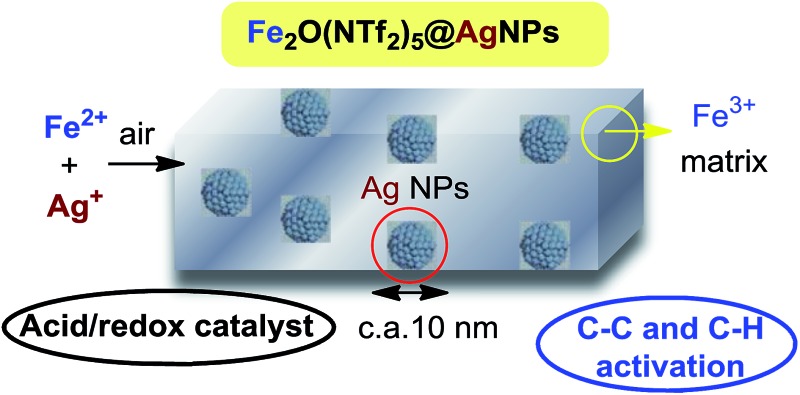
A conceptually new formation of bimetallic nanosized solids with acid and redox properties for catalytic C–C and C–H activation is presented.

## 1. Introduction

Metal triflimides M^*n*+^Tf_*n*_ [M: metal cation, Tf: ^–^N(SO_2_CF_3_)_2_] are strong Lewis acids due to the high delocalization of the negative charge in the triflimide anion, which confers high mobility to associated cations and protons. Contrary to other soft acids with highly delocalized anions (*i.e.* tetrafluoroborates and hexaflurometalates), triflimide salts are relatively stable and safe-to-handle^1^ and, for instance, LiNTf_2_ is commercially employed as an electrolyte in batteries. These properties make triflimide salts powerful Lewis acid catalysts to activate C–C bonds and, if the redox properties of the metal cation are tuned with ligands, also very active catalysts to oxidize reluctant C–H bonds.^2^ However, both acid/redox functions are typically mutually exclusive and their concomitant use in a single reaction is hampered. Besides that, triflimide salts are expensive and difficult to recover when in solution, which limits their use basically to a laboratory scale. Thus, it is clear that the synthesis of a recoverable solid triflimide catalyst, insoluble in common solvents, and with its acid/redox sites operative at the same time, would solve those problems. However, to our knowledge, no solid triflimide has been reported yet.^3,4^ Here we present a new preparation concept that allows the formation of a family of triflimide solids with very strong acidity and readily available redox metal sites. The material consists of metal (Fe^3+^, Cu^2+^, Yb^3+^ or Bi^3+^) μ-oxide triflimides on Ag nanoparticles (Ag NPs) concomitantly formed under ambient conditions, after adding thiophenol to a solution of the corresponding metal triflimide and Ag^+^. The mechanism follows a simple redox-coupled sequence to furnish self-supported solids that behave as efficient acid, redox and acid/redox heterogeneous catalysts for different C–H and C–C activation reactions.

## 2. Results and discussion

### 2.1. Synthesis, characterization and structure of the solid Fe_2_O(NTf_2_)_5_@AgNPs

**Fig. 1:**
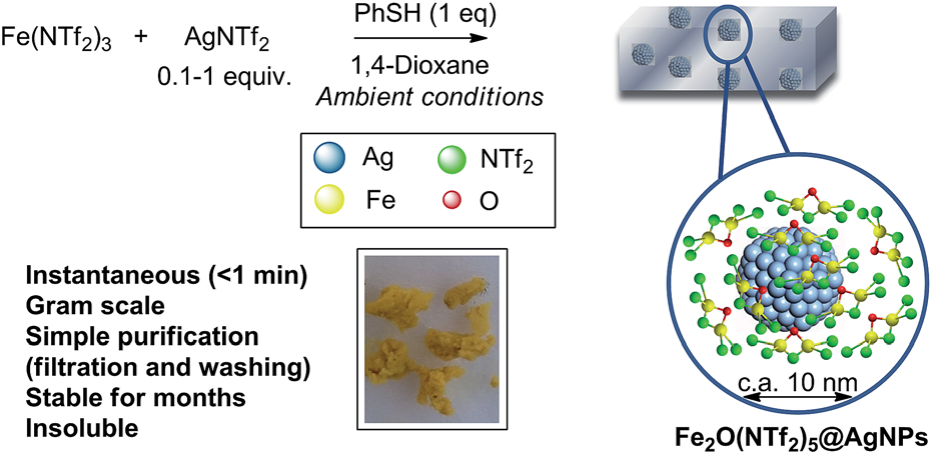
Synthesis of Fe_2_O(NTf_2_)_5_@AgNPs. Reaction conditions: Fe (NTf_2_)_3_ (6.16 mmol), AgNTf_2_ (0.1–1.0 equivalent), thiophenol (6.16 mmol), 1,4-dioxane (80 mL), room temperature, <1 min to 1 h, 28–90% yield. See details in ESI.† A photograph of the yellow solid and a model (not scaled) are also provided.

Fig. 1 shows the preparation of the solid material. The procedure consists of mixing Fe^3+^ triflimide [Fe(NTf_2_)_3_] with Ag triflimide (AgNTf_2_, 0.1–1.0 equivalent) in 1,4-dioxane at room temperature, and then adding one equivalent of thiophenol (PhSH) at once. A soft yellow solid precipitates in up to 90% yield in 2 gram scale for Ag : Fe molar ratio = 0.5.

Analysis using inductively coupled plasma-atomic emission spectroscopy (ICP-AES) gives 5.8 wt% of Fe and 6.2 wt% of Ag for the solid prepared with an Ag : Fe molar ratio = 0.5, which accounts for the amounts of Fe and Ag atoms initially added. Elemental analysis (CHNS) of the solid gives a sulfur-to-nitrogen ratio of 2 : 1, that corresponds exactly to triflimide anions, and that in principle discards the presence of thiophenol in the structure. Analysis of the organic molecules present in the solid after dissolution in HNTf_2_, extraction with diethyl ether and quantification using gas chromatography-mass spectrometry (GC-MS) with *n*-dodecane as an external standard, confirms the lack of thiols and shows the presence of ∼15 wt% of 1,4-dioxane. Thermogravimetric (TG) analysis (Fig. S1 in ESI†) confirms the amount of 1,4-dioxane present (16 wt%, loss at ∼100 °C) and also shows that the material contains 4 wt% of water and two types of triflimide anions, that desorb at ∼200 °C (10 wt%) and 300 °C (47 wt%). With all these data in hand, we can calculate the initial formula Ag_0.5_Fe(NTf_2_)_2.5_·(dioxane)_2–3_·H_2_O_1–2_ for the solid material.

**Fig. 2:**
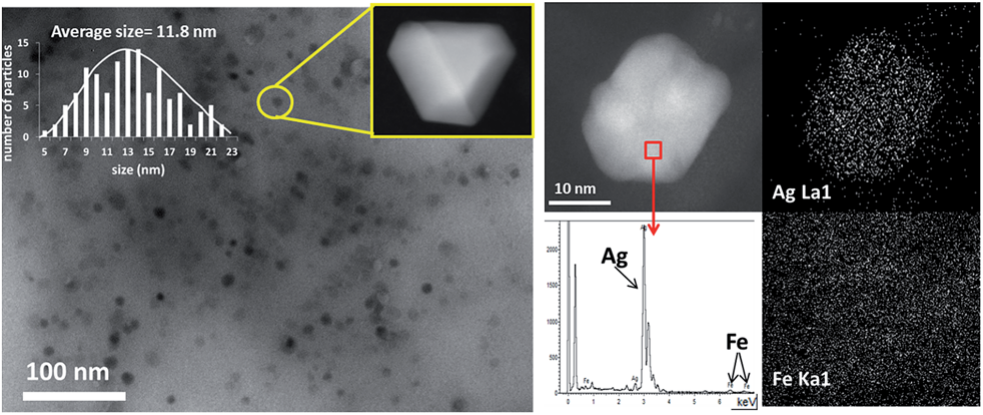
Left: a transmission electron microscopy (TEM) photograph of the Fe_2_O(NTf_2_)_5_@AgNPs solid (Ag : Fe mol ratio = 0.5) where well-dispersed Ag nanoparticles (∼12 nm average size) can be appreciated, with the corresponding histogram for at least 5 different photos and 150 particles. The inset shows a scanning transmission electron microscopy-dark field (STEM-DF) photograph of a well-faceted Ag NP. Right: scanning transmission electron microscopy-dark field (STEM-DF) micrograph of an Ag NP with the corresponding electron-dispersive X-ray (EDX) analysis of the squared area and the mapping for Ag (top) and Fe (bottom).

Fig. 2 shows transmission electron microscopy (TEM) photographs of the material (Ag loading 5.1 wt%, 0.5 equivalents of starting Ag) and well-dispersed, crystalline Ag NPs can be seen. Electron-dispersive X-ray (EDX) analysis indicates that the analyzed area of the NP is mainly Ag (98.5%), and mapping of Ag and Fe atoms in the whole micrograph shows that, while all Ag atoms are concentrated in the NP, Fe atoms are dispersed throughout the micrograph. On average, the material presents the expected molecular Ag : Fe mol ratio (0.5) and the photographs suggest that the Ag NPs are somehow embedded within a matrix of Fe.

**Fig. 3:**
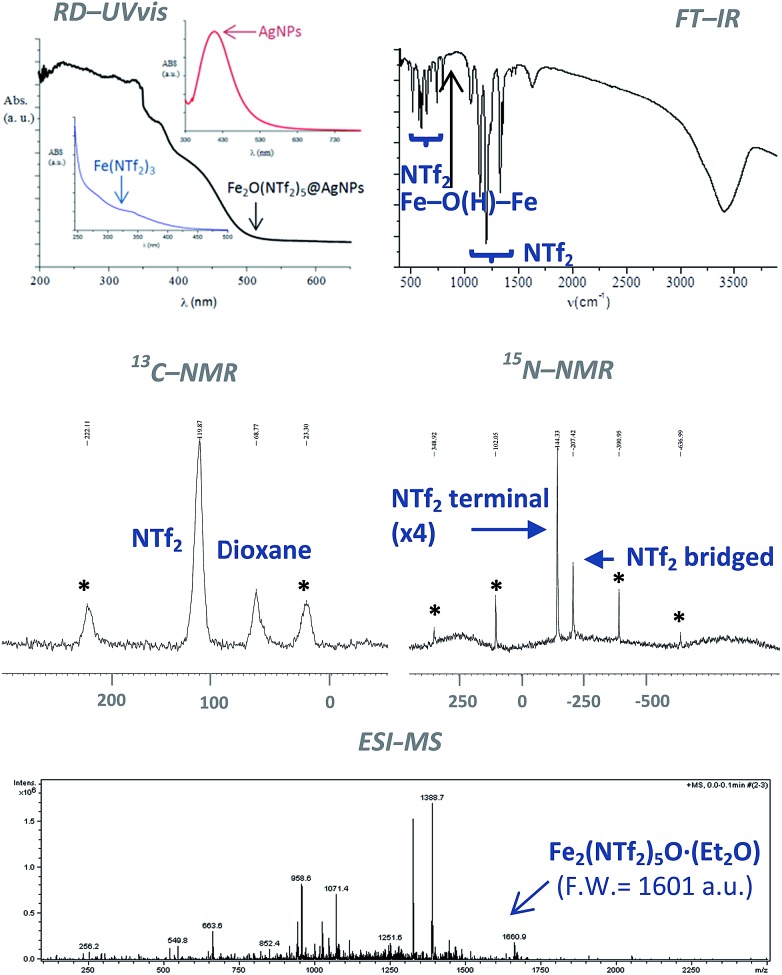
Top: Fourier transform-infrared (FT-IR) and diffuse reflectance ultraviolet-visible (RD-UV vis) spectra of the Fe_2_O(NTf_2_)_5_@AgNPs solid. For the sake of comparison, the UV-vis spectra of Fe(NTf_2_)_3_ and 10 nm Ag nanoparticles in solution are also shown. Middle: ^13^C and ^15^N nuclear magnetic resonance-magic angle spinning (NMR-MAS) spectra of the ^15^N isotopically-labelled yellow solid (* = rotational peaks). Bottom: electrospray ionization mass spectrum (ESI-MS) of the solid extracted with diethyl ether.

The classical analytical test for Fe^2+^ with ICl does not give any trace of Fe^2+^ in the solid after dissolution with HNTf_2_. In contrast, the analytical test with SnCl_2_ gave Fe^3+^ as the only iron species present in the solid. The diffuse reflectance ultraviolet-visible (RD-UV vis) spectrum of the solid shown in Fig. 3 (top-left) confirms the absence of d–d transition bands for low spin Fe^2+^ complexes, that should appear at around 600 nm. These results indicate that Fe^3+^ is the main iron species present in the solid, with less than 0.1–0.01% of Fe^2+^. Notice that the RD-UV vis spectrum fits well with the sum of the individual absorption spectra of independently-prepared Fe(NTf_2_)_3_ and AgNPs (see ESI†).

Fig. 3 (top-right) shows the Fourier transform-infrared (FT-IR) spectrum of the material with the presence of two different triflimide peaks (see Table S1 in ESI† for a complete list of bands and discussion), in accordance with the TG analysis. The triflimide anions are coordinated to Fe cations either through the oxygen or nitrogen atoms of the triflimide groups.^5^ The IR spectrum also reveals that a μ-hydroxo or μ-oxo bridge between two Fe atoms is present.^6^ These results indicate that the solid is formed by oxo-bridged Fe_2_O(NTf_2_)_5_ species.

Fig. 3 (middle) shows the ^13^C nuclear magnetic resonance-magic angle spinning (NMR-MAS) spectrum of the solid, with the expected peaks for 1,4-dioxane (∼69 ppm) and triflimide (∼120 ppm). Since no differentiation by ^13^C NMR can be expected for different triflimides, we prepared a sample of isotopically-labelled ^15^N-solid, *i.e.* Fe_2_O(^15^N-Tf_2_)_5_@AgNPs, by using Fe(^15^NTf_2_)_3_ and Ag^15^NTf_2_ as starting metal salts during the synthetic procedure depicted in Fig. 1 (see Fig. S2 in ESI† for details). The corresponding ^15^N MAS-NMR spectrum in Fig. 3 shows two well-resolved ^15^N NMR peaks, one main peak that corresponds to triflimide anions bound to one Fe^3+^ atom (–144 ppm), and a second peak upshifted –63 ppm that integrates for ¼ of the former. This result is in good agreement with the existence of two different triflimide anions as indicated by FT-IR and TG measurements. Moreover, the 4 : 1 ratio fits with the μ-hydroxo or μ-oxo bridged Fe_2_O(NTf_2_)_5_ species.

To obtain direct evidence for the presence of the dimeric species, an exhaustive extraction of the material was performed in different solvents, and we found that treatment of the solid in hot diethyl ether for 3 days gives a solution that contains one single compound in amounts large enough to be analysed using electrospray ionization mass spectrometry (ESI-MS). The mass obtained for this compound is 1660.9 Daltons, as shown in Fig. 3, which can be assigned to Fe_2_(NTf_2_)_5_O·(Et_2_O) (F.W. = 1601 D). Matrix-assisted laser-desorption/ionization coupled to a time-of-flight mass spectrometer (MALDI-TOF-MS) confirms that no heavier species up to 40 000 *m*/*z* are present in this solution. These results indicate that the solid contains significant amounts of Fe_2_O(NTf_2_)_5_ species.

**Fig. 4:**
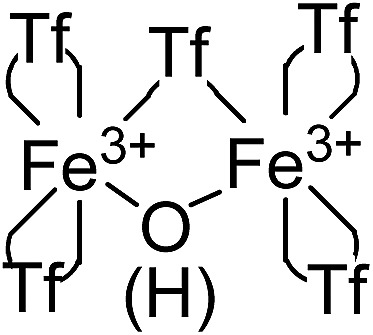
Structure of the Fe^3+^-triflimide (Tf) dimer in the solid. Coordination of the triflimides can occur through oxygen or nitrogen atoms.

Electronic paramagnetic resonance (EPR) measurements of the solid showed a lack of EPR signals for Fe in the solid (Fig. S3 in ESI†) which can only be explained by either very strong antiferromagnetic coupling between two Fe^3+^ atoms, or because the Fe centers are low spin Fe^2+^.^7^ The latter can be rejected since the material does not present any Fe^2+^ according to the analytical tests and RD-UV vis spectroscopy (*vide supra*). Thus, the fact that the material is diamagnetic at room temperature (EPR silent and no alteration in the NMR shifts) can only be explained by a strong antiferromagnetic coupling between two oxo-bridged Fe^3+^ atoms. Indeed, a semi-quantitative empirical correlation gives a *J* = 1.753 × 10^12^e^–12^: 663*R*, where *R* is half of the shortest exchange pathway, equivalent to the Fe–O distance, and agrees well with O-bridging between two Fe^3+^ atoms. The existence of a μ-hydroxo or μ-oxo bridge between two Fe^3+^ atoms explains the 5 triflimides in 4 : 1 ratio observed in the TG, FT-IR and ^15^N MAS-NMR techniques: 4 triflimides bound to a single Fe atom (two per Fe atom) and the fifth one bridging the two Fe^3+^ atoms, which close the hexagonal coordination sphere of each Fe^3+^ in the dimer Fe_2_(NTf_2_)_5_O(H), as shown in Fig. 4. Notice that, electronically, the Fe^3+^ dimer with 5 triflimides is compensated with the μ-hydroxo bridge, however, a μ-oxo bridge could also exist if the H^+^ remains associated to 1,4-dioxane or water molecules.

With all these data in hand, we can say that the structure of the yellow solid obtained is formed by well-dispersed Ag nanoparticles embedded in a matrix of O, triflimide-dibridged Fe^3+^ dimers, named hereafter as Fe_2_(NTf_2_)_5_O@AgNPs and depicted in Fig. 1. The reasonable dispersion of Ag NPs in the solid must be related to the very fast (<1 min) formation of the material, which prevents further agglomeration, giving finally Ag NPs without the aid of any ligand or additional support.^8^

### 2.2. Catalytic results of Fe_2_O(NTf_2_)_5_@AgNPs

#### 2.2.1. Acid-catalysed C–C activation

**Fig. 5:**
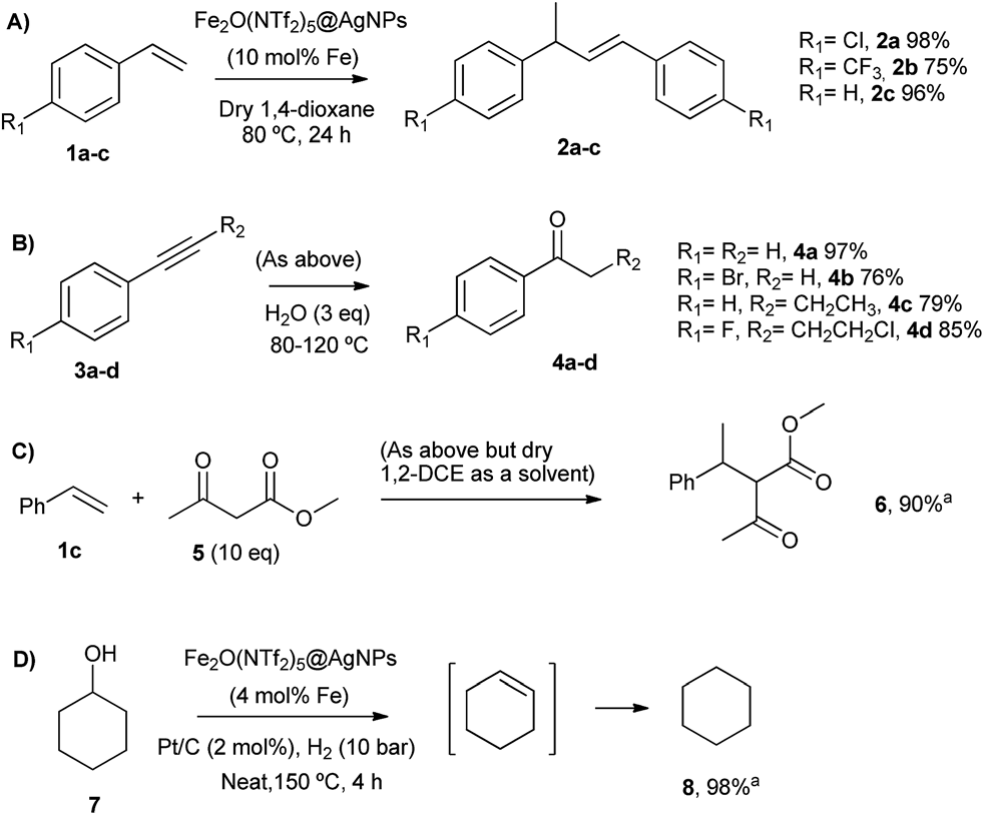
Acid-catalysed reactions with Fe_2_(NTf_2_)_5_O@AgNPs. Isolated yields. From top to bottom: (A) head-to-tail dimerization of styrenes, (B) Markovnikov hydration of alkynes, (C) addition of methyl acetoacetate to styrene, and (D) hydrodeoxygenation of cyclohexanol. ^a^GC yield.

Taking into account the potential strong acidity of the solid, its catalytic activity was tested for reactions that require an acidity value *H*_0_ < –12, *i.e.* reactions that can only be performed with concentrated H_2_SO_4_ or stronger acids. The catalytic solid material was that directly obtained from the synthetic procedure described above (*i.e.* precipitated, washed in hexane and dried under vacuum), and no grinding or sieving was needed to achieve reproducible results. Fig. 5 shows the results obtained for the different acid-catalysed reactions.

The head-to-tail dimerization of styrenes (Fig. 5A) is a useful reaction to obtain branched styrenes in a 100% atom-economical manner,^9^ and it has been catalyzed previously with, among others, Pd triflate complexes,^10^ a combination of metal complexes such as Pd/In triflate/phosphines^11^ or Co/Zn,^12^ and with Fe(NTf_2_)_3_,^13^ all of them in homogenous solution. Thus, a simple solid catalyst for this reaction remains a challenge. Indeed, only the linear (not branched) dimerization of styrenes has been reported with a heterogeneous catalyst, *i.e.* Ru supported on CeO_2_, and using formaldehyde or ethanol as promoters to give a 63% yield of 1,4-diaryl-1-butenes.^14^ Fe_2_(NTf_2_)_5_O@AgNPs catalyzes the regioselective head-to-tail dimerization of styrenes **1a–c** to **2a–c** in yields up to 98%. The hot filtration test for styrene **1a** showed that the catalytic activity completely stops when the solid catalyst is filtered off, which confirms the heterogeneous nature of the catalysis.

The Markovnikov hydration of alkynes (Fig. 5B) was earlier catalysed by Hg salts at the industrial level^15^ and, due to toxicity issues, alternative catalysts based on transition metals are currently being explored, mainly gold.^16–18^ It is difficult to find efficient catalysts that operate under mild conditions beyond noble metals,^7^ and systems based on Brönsted acids, either homogeneous or heterogeneous, require high wt% loadings, harsh reaction conditions (>150 °C) or very particular modifications of the catalytic system (additives, singular reaction media such as microemulsions, and surface modifications in solids).^19–21^ Fe_2_O(NTf_2_)_5_@AgNPs catalyzes regioselectively the hydration of alkynes **3a–d** to ketones **4a–d** without any additive in up to 97% yield.

The addition of methyl acetoacetate to styrenes (Fig. 5C) was originally reported with Fe complexes in stoichiometric amounts^22^ and further developed with noble metal catalysts,^23^ until Fe could be employed catalytically.^24^ It is not easy to find a heterogeneous catalyst for this reaction. Fe_2_O(NTf_2_)_5_@AgNPs is able to catalyze the reaction between styrene **1c** and methyl acetoacetate **5** to give the product **6** in 90% yield.

The hydrodeoxygenation reaction (Fig. 5D) is of interest in the biorefining industry to obtain alkanes from oxygen-rich biomass derived chemicals.^25^ For that, a catalytic combination of a soluble very acidic metal salt, such as a triflate, and a solid hydrogenation catalyst, such as Pt/C, is employed.^26^ Thus, it would be desirable to have both functions on a solid, either as a single solid or as a composite. A catalytic composite of Pt on TiO_2_/C has been reported^27^ and operates at temperatures of ∼300 °C. Here, a composite of Fe_2_O(NTf_2_)_5_@AgNPs and Pt/C catalyzes the hydrodeoxygenation of cyclohexanol **7** to cyclohexane **8** in 98% yield at 150 °C.

In summary, Fe_2_O(NTf_2_)_5_@AgNPs catalyzes the synthetically useful, acid strength-demanding reactions shown above in very good yields, with similar catalytic activity to the state-of-the-art soluble catalysts (Table S2†). For the sake of comparison, the dimerization of styrene **1a** and the hydration of phenylacetylene **3a** were also carried out with representative strong solid acid catalysts such as Nafion™,^28^ H-USY zeolite,^29^ and sulfated zirconia,^30^ under the indicated conditions (Fig. S4†). The results showed that only Nafion™ improves on the catalytic activity of Fe_2_(NTf_2_)_5_O@AgNPs, while H-USY and sulfated zirconia are much poorer acid catalysts, which places Fe_2_O(NTf_2_)_5_@AgNPs among the most effective solid acid catalysts reported.

#### 2.2.2. Redox-catalysed C–H activation

Selective oxidation of C–H bonds with environmentally benign reagents such as O_2_ or H_2_O_2_ under mild reaction conditions is one of the main challenges in organic synthesis.^2^ Nature makes use of enzymes with non-heme O-bridged bisiron centers,^31^ and successful bio-mimetic lines of research have been reported with O-bridged bisiron complex catalysts having low-coordinating ligands for C–H activation and C–C cleavage.^32–35^ Given the resemblance between these adducts^32,33^ and the Fe^3+^ dimer present in Fe_2_O(NTf_2_)_5_@AgNPs (see Fig. 4 above), we tested Fe_2_O(NTf_2_)_5_@AgNPs as a catalyst for the oxidation of alkanes under similar conditions to those reported, but without the need of adding AcOH to activate the iron catalyst.^34,35^

**Fig. 6:**
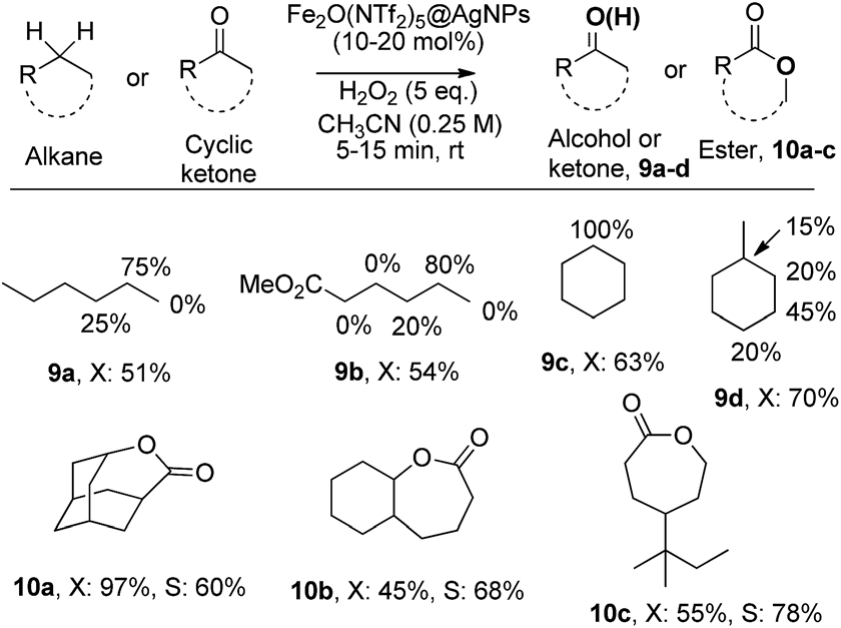
Redox-catalyzed selective CH_2_ and Baeyer–Villiger oxidations with Fe_2_O(NTf_2_)_5_@AgNPs (10 and 20 mol% of Fe for alkanes and ketones, respectively). *X*: conversion. *S*: selectivity. GC results.

The results in Fig. 6 show that Fe_2_O(NTf_2_)_5_@AgNPs converts linear and cyclic alkanes to a mixture of alcohols and ketones **9a–d**, in reasonable conversions after a few minutes at room temperature, with good selectivity towards sterically accessible methylene groups. The solid catalyst is suitable not only for methylene oxidation but also for the Baeyer–Villiger oxidation of cyclic ketones to the corresponding cyclic esters **10a–c**,^36^ under the same reaction conditions.

#### 2.2.3. Acid/redox-catalysed C–C and C–H activation

**Fig. 7:**
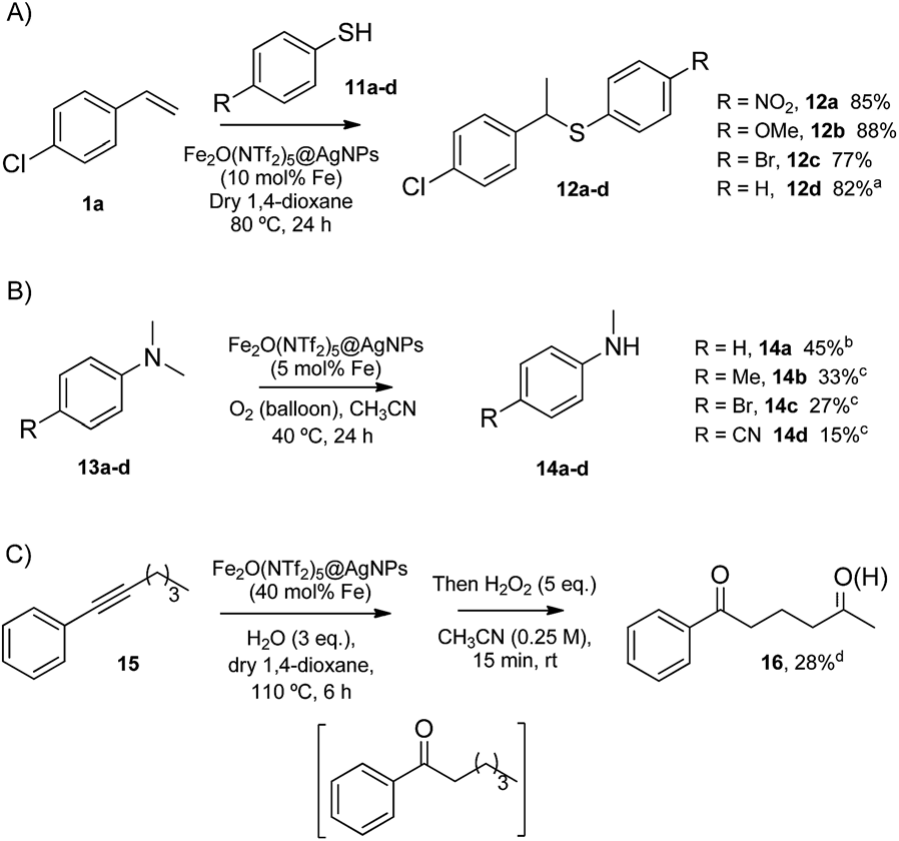
Acid/redox reactions catalysed by Fe_2_(NTf_2_)_5_O@AgNPs. Isolated yields. From top to bottom: hydrothiolation of styrenes, demethylation of *N*,*N*-dimethylanilines, and one-pot hydration of alkyne 15-CH_2_ oxidation. ^a^After 8 uses of Fe_2_(NTf_2_)_5_O@AgNPs. ^b^Combined yield with the corresponding trimers. ^c^Combined yield with the corresponding formamides. ^d^GC yield.

The performance of redox catalysis in acidic media gives access to otherwise elusive reactions. Since Fe_2_O(NTf_2_)_5_@AgNPs is operative in acid and redox reactions, separately, the catalytic activity of Fe_2_O(NTf_2_)_5_@AgNPs was tested for reactions that require both functions present (see Table S2† for comparison with state-of-the-art catalysts). The results are shown in Fig. 7.

The Markovnikov hydrothiolation of styrenes is catalyzed by a Fe^3+^/Fe^2+^ manifold mechanism with the participation of protons in the redox catalytic cycle.^37^ The results obtained (Fig. 7A) show that Fe_2_O(NTf_2_)_5_@AgNPs is as active as the state-of-the-art soluble catalysts,^38^ with good yields for the representative products **12a–d**. Besides, the solid catalyst could be reused up to 9 times after simple filtration, without a significant loss of its inherent catalytic activity (Fig. S5†).

The selective demethylation of *N*,*N*-dimethylanilines is a biomimetic reaction catalyzed by iron complexes in the presence of strong oxidants, such as PhIO.^39^ Nature makes use of O_2_ as the terminal oxidant, thus the synthetic use of O_2_ as an environmentally-friendly oxidant for this reaction would be a significant advance. Recently, it has been reported that this reaction can moderately occur under aerobic conditions (∼40% combined yield with trimers and formamides) if a metal triflate salt, *i.e.* Zn^2+^, Ba^2+^ or Y^3+^, is used in combination with the Fe^3+^ complex to form a triflate bridge between the two metal cations.^40^ It was envisioned that the solid triflimide composed of Fe dimers could mimic this system and catalyze the demethylation of *N*,*N*-dimethylanilines under similar aerobic conditions (Fig. 7B). Indeed, the reaction worked well with Fe_2_O(NTf_2_)_5_@AgNPs, with similar yields and better selectivity for the products than with the soluble metal complex since, presumably, the higher acidity of the triflimide solid overrides undesired biphenyl formation by the non-acidic aryl/Fe electron transfer.^40^

The possibility of engaging the hydration of the aryl alkyl alkyne **15** and the methylene oxidation reaction in cascade^41^ was also tested, with Fe_2_O(NTf_2_)_5_@AgNPs as a single catalyst for both processes (Fig. 7C). The result shows that the solid triflimide indeed preserves its redox catalytic activity after acting as an acid catalyst, and the 1,5-diketone **16** is thus formed in one-pot. Notice that the regioselective formation of the first carbonyl group by acid-catalyzed hydration directs the later oxidation of the methylene group.

#### 2.2.4. Making the Ag NPs accessible to reactants

When the solid with an Ag : Fe ratio = 0.5 was tested for reactions typically catalyzed by Ag NPs of ∼10 nm, including the epoxidation of styrene,^42^ the aerobic dehydrogenation of alcohols^43^ and the synthesis of azocompounds from anilines (see Fig. S6 in ESI†),^44^ no conversion was found for any of these reactions. Blank experiments with independently synthesized Ag NPs of ∼10 nm showed the expected reactivity for the Ag catalyst.^42–44^ Therefore, the lack of catalytic activity of the Ag nanoparticles in the Fe_2_O(NTf_2_)_5_@AgNPs solid suggests that the contact between Ag NPs and the reactants may be hampered in some way. To further assess if the Ag NPs in the solid were accessible to the reactant molecules, *in situ* low-temperature FT-IR experiments with carbon monoxide (CO), as a probe molecule, were carried out (Fig. S7 in ESI†). The results show that there is no interaction between CO and Ag^0^ or Ag^*δ*+^ atoms,^45^ which unequivocally indicates that the Ag atoms in the solid are not accessible to external chemicals. The lack of interaction between CO and Fe^3+^ was expected due to the low-coordinating nature of the triflimide anions and reflects the high acidity of the Fe^3+^ atoms in the solid. Considering the amount and size of Ag NPs in the material for Ag : Fe ratios ≤ 0.5 and the number of Fe atoms, a simple calculation shows that the number of Fe dimers is at least 10^4^ higher than the number of Ag nanoparticles. Thus, there are enough Fe_2_O(NTf_2_)_5_ molecules to embed all the NPs. In fact, only 5% of Fe dimers are enough to cover the whole surface of the Ag NPs. It appears then that the Fe^3+^ matrix acts as a chemical shield for the Ag NPs, conferring them protection against air, water or any other external atmosphere. For instance, the material (Ag : Fe mol ratio = 0.5) is stable after heating at 200 °C for 1 day under an H_2_ atmosphere of 10 bars and no agglomeration of Ag NPs was observed after this treatment.

**Fig. 8:**
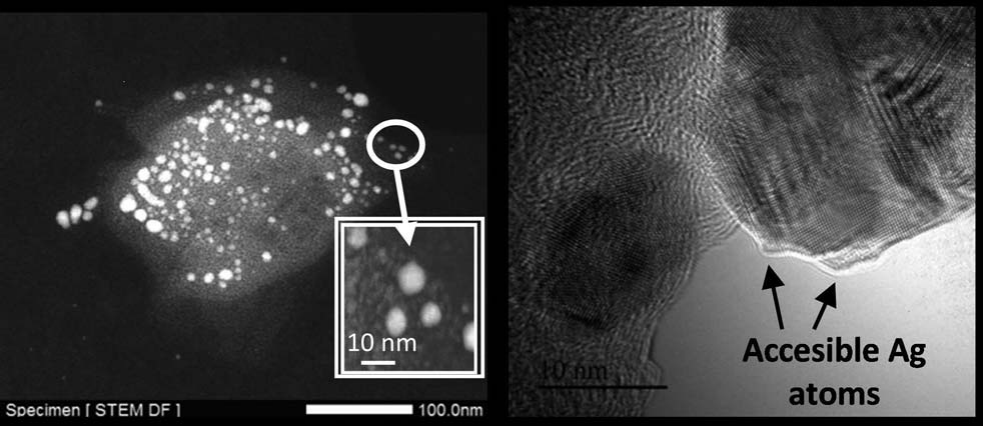
Scanning transmission electron microscopy-dark field (STEM-DF, left) and high-resolution transmission electron microscopy (HR-TEM, right, black bar accounts for 10 nm) photographs of Fe_2_O(NTf_2_)_5_@AgNPs synthesized with a Ag : Fe^3+^ mol ratio = 1. The inset magnifies a particular area (inverted).

However, it should be in principle possible to make accessible the Ag NPs in the solid by just increasing the Ag to Fe^3+^ ratio, in such a way that the Fe^3+^ triflimide dimers will not completely cover the Ag NPs. Fig. 8 shows that, indeed, the solid material has clearly visible ∼10 nm Ag NPs on the surface when the synthesis is performed with an Ag : Fe mol ratio = 1. The resultant material with accessible Ag NPs is active for the classical Ag-catalyzed reaction of aniline to give azobenzene.^44^ The solid catalyst now gives a 20% conversion with a turnover number (TOF) relative to the exposed Ag atoms of 100 (see Fig. S6 in ESI†). Comparatively, the Ag : Fe mol ratio = 0.5 solid is completely inactive and commercially available Ag/C gives only 7% conversion. These results confirm that the accessibility of the reactants to the Ag NPs in the solid material can be regulated by varying the relative amount of metals during the synthesis.

In summary, the results shown in Section 2.2 provide a picture of the rather unique acid/redox catalytic behavior of the Fe_2_O(NTf_2_)_5_@AgNPs solid.

### 2.3. Mechanism of formation of Fe_2_O(NTf_2_)_5_@AgNPs

**Fig. 9:**
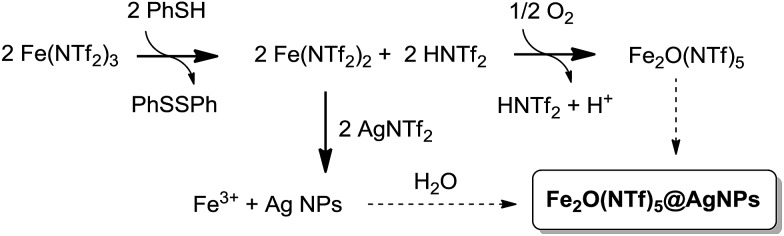
Proposed mechanism for the formation of Fe_2_O(NTf_2_)_5_@AgNPs.

A possible mechanism for the formation of the Fe_2_O(NTf_2_)_5_@AgNPs solid is depicted in Fig. 9. This mechanism is supported by reactivity tests and isotopic experiments, and it consists of the one-electron reduction of Fe(NTf_2_)_3_ to Fe(NTf_2_)_2_ by PhSH, forming a coordinatively unsaturated Fe^2+^ species that re-oxidizes at expense of reducing Ag^+^ to Ag NPs or, alternatively, with air.

The one-electron reduction of Fe(NTf_2_)_3_ to Fe(NTf_2_) is supported by the quantitative transformation of the one-electron reductant PhSH to diphenyl disulfide Ph_2_S_2_, observed using GC-MS and liquid NMR measurements of the solution, when PhSH is added to a mixture of Fe(NTf_2_)_3_ and AgNTf_2_, to form the yellow solid. In contrast, if PhSH is added to a solution of Fe(NTf_2_)_3_ without AgNTf_2_ present in the medium, the solid does not form despite Ph_2_S_2_ being quantitatively produced. Furthermore, if PhSH is added to a solution of AgNTf_2_ without Fe(NTf_2_)_3_ present, no reaction occurs. These results indicate that the one-electron reduction occurs to form Fe^2+^, in agreement with the well-known single electron transfer (SET) process between PhSH and Fe^3+^ salts at room temperature.^37^ Other potential reductants for Fe^3+^ such as isopropanol and Ce^3+^ failed to form the solid.

The yield of solid decreases significantly if an excess of PhSH with respect to metal triflimide is employed during the synthesis (90%, 65%, and 28% for 1, 3 and 10 equivalents of PhSH, respectively), due to the formation of stable Ag^+^–thiolate complexes. When Au, Pd and Pt salts were tested instead of Ag no solid formation occurred. These results, in principle, discard the possibility that thiophenol acts as a “seed” for nanoparticle formation, and the SET transfer reaction seems to be predominant in the mechanism.

The second step in the mechanism of formation of the bimetallic solid is a redox reaction between the *in situ* formed Fe^2+^ and Ag^+^, to give Fe^3+^ and Ag NPs, as expected from their respective redox potentials. Accordingly, the pH of the solution is made very acidic (<1) by the released triflimidic acid, and the protons may stay either coordinated to the oxygen atoms of the O-bridges or to the dioxane molecules.

The third and last step is the formation of the μ-oxo or μ-hydroxo bridge between two Fe^3+^ atoms. To shed light on this step, we used an atmosphere of ^18^O_2_, and the corresponding FT-IR spectrum of the yellow solid showed the formation of an Fe–^18^O–Fe bridge according to the slight shift of the corresponding IR band with respect to the non-labelled yellow solid (see Fig. 3).^6^ Additionally, we found that H_2_^18^O is formed in solution, as determined by CG-MS. These results support that Fe^2+^ oxidizes to Fe^3+^ not only with Ag^+^ but also with the O_2_ present in the atmosphere, and that O_2_ is then reduced to H_2_O. This reaction complements the oxidation with Ag^+^ and closes the mass balance. The bridged oxygen atom would then come from reduced molecular oxygen, either directly or after reduction to H_2_O. To test this last possibility, H_2_^18^O was added to an anhydrous solution of triflimides prior to the formation of the solid under air. As occurred previously when working with an ^18^O_2_ atmosphere, the formation of the isotopically-labelled Fe_2_^18^O(NTf_2_)_5_@AgNPs was observed, with the concomitant consumption of H_2_^18^O observed using GC-MS. These results support that the oxygen atom for the O-bridged Fe^3+^ dimer comes from water molecules present in the medium. If so, the bridge should also be produced under an inert atmosphere provided that water is in the medium. Indeed, a 65% yield of solid was obtained under N_2_ when 0.5 equivalents of Ag were used. It must be noticed, however, that the formation of the bridge is less efficient in the absence of O_2_ since a 90% yield is obtained when the process is run aerobically, which is explained by the better oxidation of Fe^2+^ to Fe^3+^ when molecular oxygen is present.

All the steps described above occur in less than 1 minute at room temperature, and the formation of a coordinatively unsaturated Fe(NTf_2_)_2_ species seems to play a key role in the formation of the solid, accommodating the bridging NTf_2_ and O species. In agreement with this, when a sample of independently prepared Fe^2+^(NTf_2_)_2_ was used as the starting material to form the solid,^13^ with the whole hexacoordination sphere saturated (2 bridging triflimides and 4 bisoxo-coordinated terminal triflimides, 2 per Fe^2+^ atom), no solid was formed. This result evidences the need for *in situ* reducing the Fe^3+^ triflimide.

### 2.4. Extension of the work to other metallic systems: synthesis of Cu, Bi and Yb-triflimide@AgNPs

According to the mechanism in Fig. 9, it might be expected that other metal cations could participate in the proposed redox sequence, as long as the corresponding metal triflimide can suffer the SET reduction with PhSH and the generated reduced metal cation is re-oxidized back with O_2_ and/or Ag^+^. If so, a family of ligand-free, self-supported bimetallic solids could be available following the preparation method described here. After examining the redox potentials of tabulated metal cations and testing those suitable, we found that the triflimides of Cu^2+^, Yb^3+^ and Bi^3+^ were able to form the corresponding solids in moderate to good yields (84% for copper, 75% for bismuth and 63% for ytterbium) under the same reaction conditions as for Fe_2_O(NTf_2_)_5_@AgNPs. Other cations such as Ru^3+^ or Co^3+^ failed to produce the corresponding solids.

**Fig. 10:**
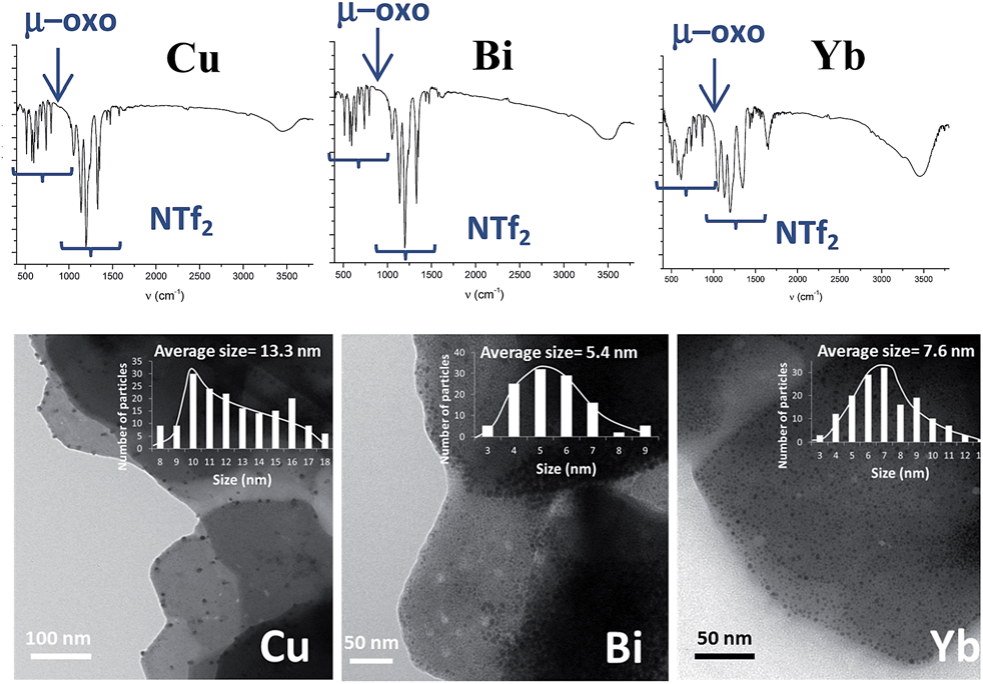
Top: FT-IR spectra of the solids made by the same procedure as Fe_2_O(NTf_2_)_5_@AgNPs but with Cu(NTf_2_)_2_, Bi(NTf_2_)_3_ and Yb(NTf_2_)_3_ as starting materials, from left to right, respectively. Bottom: transmission electron microscopy (TEM) photographs with the corresponding histograms for, at least, 5 different photos.

Fig. 10 shows the characterization of the solids using FT-IR and TEM. The results suggest that, in principle, these solids have a similar structure to Fe_2_O(NTf_2_)_5_@AgNPs, *i.e.* they are formed by well-dispersed Ag NPs embedded within a metal triflimide matrix. In the case of Cu, the Ag NPs flourish to the surface (see left microphotograph in Fig. 10).

**Fig. 11:**
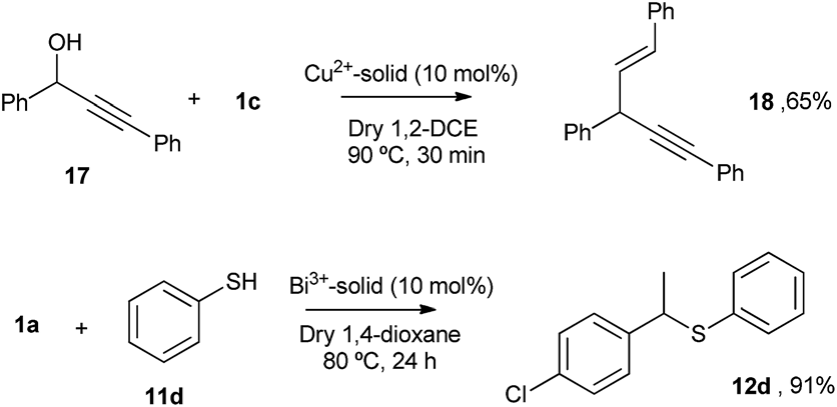
Top: vinylation of 1,3-diphenylpropargyl alcohol **17** with styrene **1c** catalyzed by the Cu-triflimide@AgNPs solid. Bottom: hydrothiolation of 4-chlorostyrene **1a** catalyzed by the Bi-triflimide@AgNPs solid. GC yields.

The Cu and Bi materials were tested as catalysts in the vinylation of 1,3-diphenylpropargyl alcohol^46–48^ and in the hydrothiolation of styrenes,^37^ respectively. Fig. 11 shows that products **18** and **12d** were obtained in good yields, similar to those previously reported.

## 3. Conclusions

The direct synthesis of self-supported triflimide metal matrices on Ag nanoparticles has been accomplished by a conceptually-new procedure that involves a redox-coupled sequence of two metal salts under air.^49,50^ This straightforward, sustainable and high-scale method of preparation of a catalytic triflimide solid opens a new way of synthesizing strong solid acids with redox sites. The nanosized bimetallic solids here formed can act as an acid catalyst, a redox catalyst or a bifunctional acid/redox catalyst.
