# Supplementary material for: Bimetallic nanosized solids with acid and redox properties for catalytic activation of C–C and C–H bonds
Source: Chem Sci. 2016 Aug 26;8(1):689–96. doi: 10.1039/c6sc03335k (PMC5297923; doi:10.1039/c6sc03335k)
Supplement: Supplementary file 1 [file SC-008-C6SC03335K-s001.pdf]

Electronic Supplementary Material (ESI) for Chemical Science.
This journal is © The Royal Society of Chemistry 2016

ELECTRONIC SUPPLEMENTARY INFORMATION

Bimetallic nanosized solids with acid and redox properties for catalytic activation of C–C and C–H bonds

Jose R. Cabrero-Antonino,[a] María Tejeda-Serrano,[a] Manuel Quesada,[a] Jose A. Vidal-Moya,[a] Antonio Leyva-Pérez,[a]* and Avelino Corma.[a]*

[a] Instituto de Tecnología Química. Universitat Politècnica de València-Consejo Superior de Investigaciones Científicas. Avda. de los Naranjos s/n, 46022, Valencia, Spain.

Corresponding authors: acorma@itq.upv.es, anleyva@itq.upv.es

Phone: +34963877800; Fax: +349638 77809.

TABLE OF CONTENTS:

- Experimental section:
  - General……………………..………………S2
  - Reaction Procedures……………………......S2
  - Characterisation ………………………………S10
- Figures S1-S7..……………………..………………S18
- Tables S1-S2...……………………..………………S22
- Scheme S1……………………………………...…..S24
- NMR spectra……………………………………….S25

Experimental Section

General

Reagents and solvents were obtained from commercial sources (Aldrich) and were used without further purification otherwise indicated. All the products obtained were characterised by GC-MS, ^{1}H- and ^{13}C-NMR, and DEPT. When available, the characterisation given in the literature was used for comparison. Gas chromatographic analyses were performed in an instrument equipped with a 25 m capillary column of 5% phenylmethylsilicone. GC/MS analyses were performed on a spectrometer equipped with the same column as the GC and operated under the same conditions. ^{1}H, ^{13}C and DEPT were recorded in a 300 MHz instrument using $CDCl_3$ as solvent containing TMS as an internal standard. Elemental analyses of the solids and some products were determined by chemical combustion using a CHNSO analyzer. Solid IR spectra of the compounds were recorded on a Jasko 460 plus spectrophotometer by previous mixture with KBr. IR spectra of the liquids were recorded on a Jasko 460 plus spectrophotometer by impregnating the windows with a dichloromethane solution of the compound and leaving to evaporate before analysis. IR peaks are defined as very intense (vi), intense (i), medium (m), low (l) and broad (br). Absorption spectra were recorded on an UV/Vis spectrophotometer (UV0811M209, Varian). Transmission or scanning electron microscopy measurements were carried out in a JEOL instrument.

Reaction Procedures

Synthesis of the solid $Fe_2O(NTf_2)_5$@AgNPs (Figure 1). $FeCl_3$ (1.0 g, 6.16 mmol, Aldrich, > 98%) and $AgNTf_2$ (7.39 g, 18.48 mmol, Aldrich, 97%) were placed in a 250 mL cylindrical vial and 1,4-dioxane (80.0 mL) was added. The mixture was magnetically stirred at room temperature for 30 min. After this time, the precipitation of silver chloride in a dark brown solution was observed. Then, the reaction mixture was passed through a microfilter syringe for the elimination of the solids and transferred to a new cylindrical vial containing $AgNTf_2$ (0.1-1.0 eq respect to iron, Aldrich, 97%). Thiophenol (652 μL, 6.16 mmol, Aldrich, 97%) was

added and a yellow solid precipitated immediately. The solid was washed with *n*-hexane (3x 50.0 mL) and dried under vacuum to give $Fe_2O(NTf_2)_5$@AgNPs (4.23 g, 90% yield).

Synthesis of ^{15}N-benzyl sulfonamide (Figure S2). DIPEA (2.46 mL, 14.18 mmol) was added to a solution of ^{15}N-benzylamine (776.5 µL, 7.1 mmol) in dry DCM (70 mL) under nitrogen atmosphere. The reaction mixture was cooled down to -78 ºC, and triflic anhydride (5.0 g, 17.73 mmol) was added dropwise. The reaction mixture was slowly warmed up to room temperature during 1 h and stirred at this temperature for 1 h. Then, aqueous HCl (3 %) was added. The aqueous phase was extracted with DCM and the combined organic layers were dried over $MgSO_4$. Filtration and evaporation of the solvent left a crude mixture, which was refluxed in pentane. Before cooling down to room temperature, the pentane phases were collected, repeating this operation several times. Evaporation of the pentane fractions left ^{15}N-benzyl sulfonamide as a pale brown solid (2.17 g, 83 %).

Synthesis of ^{15}N-silver(I) triflimide (Figure S2). ^{15}N-benzyl sulfonamide (1.19 g, 3.20 mmol) was dissolved in ethanol (16 mL) and stirred during 8 h at room temperature. The volatiles were evaporated from the reaction mixture under reduced pressure at 60 ºC. The oil residue was dissolved in dry toluene (25 mL). Then, silver oxide (0.371 g, 1.6 mmol) was added and the light-protected reaction mixture was heated under reflux for 3 h, after which complete dissolution of the solid was observed. The reaction mixture was cooled down, filtered over Celite® and concentrated to 1:3 of the volume. Finally, the product $Ag^{15}NTf_2$ was precipitated with pentane as a yellow hygroscopic solid (0.87 g, 70 %).

Typical reaction procedure for the synthesis of the solid $Fe_2O(^{15}NTf_2)_5$@AgNPs (Figures 3 and S2). $FeCl_3$ (100.0 mg, 0.61 mmol, Aldrich, > 98%) and $Ag^{15}NTf_2$ (717.0 mg, 1.84 mmol) were placed in a 25 mL cylindrical vial. Then, 1,4-dioxane (10.0 mL) was added. The mixture was magnetically stirred at room temperature for 30 min. After this time, the precipitation of silver chloride and the formation of dark brown solution were observed. Then, the reaction mixture was passed through a microfilter syringe for the elimination of the solid precipitate. Next, $Ag^{15}NTf_2$ (0.1-0.5 eq respect to iron) was placed and thiophenol (65.1 µL, 0.61 mmol,

Aldrich, 97%) was added dropwise and a yellow solid precipitated immediately. Then, the solid was washed with *n*-hexane (3x 5.0 mL) and dried under vacuum achieving $Fe_2O(^{15}NTf_2)_5$@AgNPs (419.0 mg, 87% yield).

Synthesis of iron(II) triflimide [$Fe(NTf_2)_2$]. Iron powder (2.58 g, 46.2 mmoles) was placed to a 100 mL round-bottomed flask containing one dissolution of $HNTf_2$ (5. 16 g, 18.36 mmoles) in deionized water (30.0 mL). Next, one refrigerant was adapted and the mixture was magnetically stirred at reflux for 3 days. The residue solid obtained was drying under vacuum at 150 ºC during 20 h, and the final product was obtained as a brown solid (3.98 g, 70%).

Typical procedure for UV-Vis measurements for $Fe(NTf_2)_3$ (Figure 3). 0.1 mM 1,4-dioxane solution of $Fe(NTf_2)_3$ was prepared as follows: $FeCl_3$ (4.0 mg, 0.025 mmol, Aldrich, > 99.99%) and $AgNTf_2$ (30.0 mg, 0.075 mmol, Aldrich, 97%) were placed in a 2 mL vial. A rubber septum was fitted and 1,4-dioxane (1.0 mL) was added. The mixture was magnetically stirred at room temperature for 30 min observing the precipitation of AgCl. Then, the liquid was passed through a microfilter syringe, and the filtrates were diluted to 25 mL of 1,4-dioxane and then 500 μL of this solution were diluted in 5 mL of 1,4-dioxane.

Typical procedure for UV-Vis measurements for Ag NPs (Figure 3). 0.1 mM 1,4-dioxane solution of silver nanoparticles was prepared as follows: Ag NPs (2.7 mg, 0.025 mmol) was placed in a 2 mL vial and a rubber septum was fitted. Then, 1,4-dioxane (1.0 mL) was added and the mixture was magnetically stirred at room temperature for 5 min. Next, the mixture was diluted to 25 mL of 1,4-dioxane and then 500 μL of this solution were diluted in 5 mL of 1,4-dioxane.

Typical reaction procedure for isolated products in the head-to-tail dimerisation of styrenes (Figure 5). $Fe_2O(NTf_2)_5$@AgNPs (19.08 mg, 0.0125 mmol, 10 mol% Fe) was placed in a 2 mL vial and a rubber septum was fitted. Then, 1,4-dioxane (0.5 mL) and styrene **1c** (28.7 μL, 0.25 mmol, Aldrich, > 99%) were added and the mixture was placed in pre-heated oil bath at 80 ºC and magnetically stirred for 18 h. After cooling, *n*-hexane (1 mL) was added, the liquid was passed through a microfilter syringe and one aliquot (25 μL) was analyzed by GC after

addition of dodecane (11.0 μL, 0.048 mmol) as an external standard. The filtrates were purified by TLC preparative. The silica containing product was stirred at room temperature with Et_2O (30 mL) during 15 min. The liquid was filtered through a conical funnel and silica residues were washed successively with ether (3x5 mL), concentrated under reduced pressure and, after drying under vacuum, the resulting the resulting residue 1,3-diphenyl-1-butene **2c** was obtained as a clear oil (25 mg, 95 %).

Two-step synthesis of compound 3d, 1-(4-chlorobutynyl)-4-fluorobenzene (Scheme S1 and Figure 5). Step 1: 3-Butyn-1-ol (3.02 mL, 0.04 mmol) and pyridine (256 μL, 0.004 mmol) were placed in a 10 mL round-bottomed flask and the mixture was cooled in an ice-bath. Then, thionyl chloride (2.91 mL, 0.04 mmol) was added dropwise during 10 min. The flask was shaken occasionally during the addition and, after the thionyl chloride was added, the mixture was heated under reflux for 30 min. Fractional distillation of the products gave the corresponding chloride 4-chlorobut-1-yne as a yellow liquid (3.34 ml, 95 %). **Step 2:** $Pd(PPh_3)_2Cl_2$ (140.4 mg, 0.2 mmol) and CuI (76.2 mg, 0.2 mmol) were placed in a 25 mL round-bottomed flask, a rubber septum was fitted and it was purged with N_2 leaving finally a N_2 atmosphere. Then, dry THF (20 mL), Et_3N (5.56 mL, 40 mmol), 4-chlorobut-1-yne (1.048 g, 12 mmol) and 1-fluoro-4-iodobenzene (923 μL, 8 mmol) were added and the reaction mixture was magnetically stirred at room temperature for 1 h. The liquid was filtrated under gravity and washed with dichloromethane (3 x 10 mL). The filtrates were concentrated under reduced pressure and the resulting yellow oil residue was purified by flash column chromatography on silica using *n*-hexane (100 %) as an eluent. Then, fractions were concentrated under reduced pressure and, after drying under vacuum, the resulting product compound **3d** was obtained as a yellow oil (750 mg, 51 %).

Typical reaction procedure for isolated products in the hydration of alkynes (Figure 5). $Fe_2O(NTf_2)_5$@AgNPs (38.2 mg, 0.25 mmol, 10 mol% Fe) was placed in a 10 mL round-bottomed flask and a rubber septum was fitted. Then, 1,4-dioxane (1.5 mL) was added and the mixture was magnetically stirred at room temperature for 30 min. Then, phenylacetylene **3a** (56

μL, 0.5 mmol, Aldrich, 97%) and water (28 μL, 1.5 mmol) were added and the mixture was placed in a pre-heated oil bath at 80 ºC and magnetically stirred for 20 h. After cooling, n-hexane (2x10 mL) was added and the liquid was filtrated under gravity. One aliquot (25 μL) was analyzed by GC after addition of dodecane (11.0 μL, 0.048 mmol) as an external standard. The filtrates were purified by TLC preparative. The silica containing product was stirred at room temperature with Et_2O (30 mL) during 15 min. The liquid was filtered through a conical funnel and silica residues were washed successively with ether (3x5 mL), concentrated under reduced pressure and, after drying under vacuum, the resulting compound acetophenone **4a** was obtained as a yellow oil (56 mg, 97 %).

Typical reaction procedure for addition of methyl acetoacetate 5 to styrene 1c (Figure 5). $Fe_2O(NTf_2)_5$@AgNPs (19.1 mg, 0.0125 mmol, 10 mol% Fe) was placed in a 2 mL vial and a rubber septum was fitted. Then, 1,2-dichloroethane (0.5 mL), methyl acetoacetate **5** (269.7 μL, 2.5 mmoles, Aldrich, > 99%) and styrene **1c** (28.7 μL, 0.25 mmol, Aldrich, 97%) were added and the mixture was placed in pre-heated oil bath at 80 ºC and magnetically stirred for 24 h. After cooling, *n*-hexane (1 mL) was added, the liquid was passed through a microfilter syringe and was analyzed by GC after addition of dodecane (11.0 μL, 0.048 mmol) as an external standard.

Typical reaction procedure for hydrodeoxygenation of cyclohexanol 7 (Figure 5). $Fe_2O(NTf_2)_5$@AgNPs (19.1 mg, 0.0125 mmol, 20 mol% Fe) and Pt-C (20 mg, 2 mol%) were placed in a 6 mL autoclave equipped with a manometer. Then, cyclohexanol **7** (50 μL, 0. 5 mmol) was added and the vial atmosphere was purged twice with hydrogen gas, leaving finally a 10 bar hydrogen pressure. The mixture was placed in pre-heated oil bath at 150 ºC and magnetically stirred for 4 h. After cooling, dichloromethane (1 mL) was added, the liquid was passed through a microfilter syringe and analyzed by GC after addition of dodecane (11.0 μL, 0.048 mmol) as an external standard.

Typical reaction procedure for methylene oxidation or Baeyer-Villiger oxidation (Figure 6). $Fe_2O(NTf_2)_5$@AgNPs (30.0 mg, 20 mol% Fe) and H_2O_2 (37.5 μL, 2.5 equivalents) were

added to a solution of the corresponding alkanes or cyclic ketones (0.15 mmol) in CH_3CN (0.3 mL, 0.5 M) at room temperature under magnetic stirring. After 10 minutes, fresh catalyst and H_2O_2 were added (same amounts than before) and the mixture left to stir for 10 minutes more. The final mixture was filtered and analysed by GC and GC-MS.

Typical reaction procedure for isolated products in the hydrothiolation of 4-chlorostyrene 1a (Figure 7). $Fe_2O(NTf_2)_5$@AgNPs (19.1 mg, 0.0125 mmol, 10 mol% Fe) and 4-nitrothiophenol **5a** (48.5 mg, 0.25 mmol, Aldrich, 80%) were placed in a 2 mL vial and a rubber septum was fitted. Then, 1,4-dioxane (0.5 mL) and 4-chlorostyrene **1a** (30.0 μL, 0.25 mmol, Aldrich, 97%) were added and the mixture was placed in pre-heated oil bath at 80 ºC and magnetically stirred for 24 h. After cooling, *n*-hexane (1 mL) was added, the liquid was passed through a microfilter syringe and one aliquot (25 μL) was analyzed by GC after addition of dodecane (11.0 μL, 0.048 mmol) as an external standard. The filtrates were purified by TLC preparative. The silica containing product was stirred at room temperature with Et_2O (30 mL) during 15 min. The liquid was filtered through a conical funnel and silica residues were washed successively with ether (3x5 mL), concentrated under reduced pressure and, after drying under vacuum, the resulting (1-(4-chlorophenyl)ethyl)(4-nitrophenyl)sulfide **12a** was obtained as a white solid (62 mg, 85 %).

Reuse of $Fe_2O(NTf_2)_5$@AgNPs during the hydrothiolation of 4-chlorostyrene 1a (Figure 7 and S4). Following the same procedure as above, the recovered solid catalyst $Fe_2O(NTf_2)_5$@AgNPs was washed with *n*-hexane (10 mL), dried under vacuum and weighted to recalculate the amount of reactants and solvent in the next run (10 mol% catalyst). The procedure was repeated 8 times, with a consistent yield of ~82-88% in each run. After the second and eighth run, kinetic experiments were also carried out.

Typical reaction procedure for the demethylation of *N,N*-dimethylanilines (Figure 7). In a glass tube, $Fe_2O(NTf_2)_5$@AgNPs (30 mg, 0.02 mmol) was placed and a solution of *N,N*-dimethylaniline (63.4 μL, 0.5 mmol) in 5 mL of acetonitrile was added. Then, the reaction mixture was magnetically stirred at 40 ºC in a silica bath with a dioxygen balloon. The reaction

was followed by GC and TLC. For other amines, amounts were as follows: 4-*N,N*-Trimethylaniline (72.8 μL, 0.5 mmol), 4-Bromo-*N,N*-dimethylaniline (103.1 mg, 0.5 mmol), and 4-(Dimethylamino)benzonitrile (77.9 μL, 0.5 mmol).

Reaction procedure for the one–pot hydration of alkyne 15–CH_2 oxidation (Figure 7). $Fe_2O(NTf_2)_5$@AgNPs (7.6 mg, 0.05 mmol, 40 mol% Fe) was placed in a 2 mL vial and 1,4-dioxane (0.25 mL), 1-phenylhexyne **15** (19.8 mg, 0.125 mmol) and water (7 μL, 3 equivalents) were added and the mixture was placed in a pre-heated oil bath at 110 ºC and magnetically stirred for 6 h. After cooling, CH_3CN (0.75 mL, 0.25 M) and H_2O_2 (100 μL, 5 equivalents) were added to the solution and the mixture was magnetically stirred for 15 minutes. The final mixture was filtered and analysed by GC and GC-MS.

Comparison of the catalytic results of $Fe_2O(NTf_2)_5$@AgNPs with other solid acids (Figure S4). The solid acid (~10 mol% of acid sites, 20 mg for nafion™, 90 mg for H-USY zeolite and 27 mg for sulfated zirconia) was placed in a 2 mL vial and a rubber septum was fitted. Then, either 4-chlorostyrene **1a** (0.25 mmol) or phenylacetylene **3a** (0.25 mmol) and water (0.75 mmol) were added in 1,4-dioxane solution (0.5 mL), and the mixture was placed in pre-heated oil bath at 80 and 100 ºC, respectively, and magnetically stirred for 24 h. The reactions were followed by GC.

Typical reaction procedure for the epoxidation of styrene with *tert*-butyl hydroperoxide (Figure S6). $Fe_2O(NTf_2)_5$@AgNPs (5.1 wt% Ag, 105.4 mg, 0.05 mmol, 10 mol% Ag) or Ag NPs (5.38 mg, 0.05 mmol, 10 mol%) was placed in 2 mL cylindrical vial. Then, anhydrous TBHP solution 5 M in decane (400 μL, 2.0 mmol, Aldrich) and styrene **1a** (57.4 μL, 0.5 mmol, Aldrich, 97%) were added and the mixture was placed in pre-heated oil bath at reflux and magnetically stirred for 12 h. After cooling, *n*-hexane (1 mL) was added, the liquid was passed through a microfilter syringe and was analyzed by GC after addition of dodecane (11.0 μL, 0.048 mmol) as an external standard.

Typical reaction procedure for the aerobic dehydrogenation of alcohols (Figure S6). $Fe_2O(NTf_2)_5$@AgNPs (5.1 wt% Ag, 5.27 mg, 0.0025 mmol, 1 mol% Ag) or Ag NPs (0.269 mg,

0.0025 mmol, 1 mol%) and diphenylmetanol (46.7 mg, 0.25 mmol, Aldrich, 99%) were placed in 2 mL cylindrical vial. Then, dry mesytilene (0.5 mL) was added and the mixture was placed in pre-heated oil bath at 130 ºC and magnetically stirred for 15 h. After cooling, *n*-hexane (1 mL) was added, the liquid was passed through a microfilter syringe and was analyzed by GC after addition of dodecane (11.0 μL, 0.048 mmol) as an external standard.

Typical reaction procedure for the synthesis of azocompounds from anilines (Figure S6). $Fe_2O(NTf_2)_5$@AgNPs (5.1 wt% Ag, 31.62 mg, 0.015 mmol, 6 mol% Ag) or Ag NPs (1.614 mg, 0.015 mmol, 6 mol%) and KOH (15.58 mg, 0.25 mmol, Aldrich, 90%) were placed in 2 mL cylindrical vial. Then, dry DMSO (0.5 mL) and aniline (22.7 μL, 0.25 mmol, Aldrich, 99%) were added and the mixture was placed in pre-heated oil bath at 60 ºC and magnetically stirred for 24 h. After cooling *n*-hexane (1 mL) was added, the liquid was passed through a microfilter syringe and was analyzed by GC after addition of dodecane (11.0 μL, 0.048 mmol) as an external standard.

Synthesis of the copper solid (Figure 10). $CuCl_2$ (500 mg, 3.71 mmol, Aldrich, 99%) and $AgNTf_2$ (2.88 g, 7.43 mmol, Aldrich, 97%) were placed in a 150 mL cylindrical vial. Then, 1,4-dioxane (40.0 mL) was added. The mixture was magnetically stirred at room temperature for 30 min and the precipitation of silver chloride was observed. Then, the reaction mixture was passed through a microfilter syringe for the elimination of the solid precipitate and transferred to new cylindrical vial. Next, $AgNTf_2$ (0.1-0.5 eq respect to copper, Aldrich, 97%) was placed and thiophenol (325.9 μL, 3.08 mmol, Aldrich, 97%) was added dropwise and a solid precipitated immediately. Then, the solid was washed with *n*-hexane (3x 25.0 mL) and dried under vacuum achieving copper solid in 84% yield.

Synthesis of the bismuth solid (Figure 10). $BiCl_3$ (500 mg, 1.58 mmol, Aldrich, > 98%) and $AgNTf_2$ (1.84 g, 4.74 mmol, Aldrich, 97%) were placed in a 150 mL cylindrical vial. Then, 1,4-dioxane (40.0 mL) was added. The mixture was magnetically stirred at room temperature for 30 min and the precipitation of silver chloride was observed. Then, the reaction mixture was passed through a microfilter syringe for the elimination of the solid precipitate and transferred to

new cylindrical vial. Next, $AgNTf_2$ (0.1-0.5 eq respect to bismuth, Aldrich, 97%) was placed and thiophenol (167.18 μL, 1.58 mmol, Aldrich, 97%) was added dropwise and a solid precipitated immediately. Then, the solid was washed with *n*-hexane (3x 25.0 mL) and dried under vacuum achieving copper solid in 75% yield.

Synthesis of the ytterbium solid (Figure 10). $YbCl_3$ (500 mg, 1.78 mmol, Aldrich, > 99%) and $AgNTf_2$ (2.07 g, 5.34 mmol, Aldrich, 97%) were placed in a 150 mL cylindrical vial. Then, 1,4-dioxane (40.0 mL) was added. The mixture was magnetically stirred at room temperature for 30 min and the precipitation of silver chloride was observed. Then, the reaction mixture was passed through a microfilter syringe for the elimination of the solid precipitate and transferred to new cylindrical vial. Next, $AgNTf_2$ (0.1-0.5 eq respect to ytterbium, Aldrich, 97%) was placed and thiophenol (188.34 μL, 1.78 mmol, Aldrich, 97%) was added dropwise and a solid precipitated immediately. Then, the solid was washed with *n*-hexane (3x 25.0 mL) and dried under vacuum achieving bismuth solid in 63% yield.

Typical reaction procedure for vinylation of 1,3-diphenylpropargyl alcohol 17 with styrene 1c (Figure 11). $Fe_2O(NTf_2)_5$@AgNPs (19.08 mg, 0.0125 mmol, 10 mol% Fe) and 1.3-diphenylpropargyl alcohol **17** (57.85 mg, 0.25 mmoles, Aldrich, 90%) were placed in a 10 mL round-bottomed flask and a rubber septum was fitted. Then, dry 1,2-dichloroethane (2.0 mL) and styrene (28.7 μL, 0.25 mmol, Aldrich, 97%) were added and the mixture was placed in pre-heated oil bath at 90 ºC (reflux) and magnetically stirred for 30 min. After cooling, *n*-hexane (1 mL) was added, the liquid was passed through a microfilter syringe and was analyzed by GC after addition of dodecane (11.0 μL, 0.048 mmol) as an external standard.

Characterisation

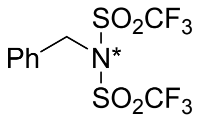

^{1}H NMR (δ, ppm; *J*, Hz): 7.53-7.45 (m, 2H), 7.44-7.36 (m, 3H), 5.09 (s, 1H). ^{13}C NMR (δ, ppm; *J*, Hz): 131.88 (C), 130.05 (CH), 129.98 (2xCH), 129.08 (2xCH), 118.96 (q, $J^{1}_{C\text{-}F}$ = 325.0, 2xCF_3), 56.79 (CH_2). ^{19}F NMR (δ, ppm): -72.12 (s, 2xCF_3).

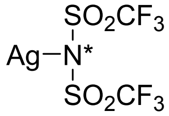

IR (ν, cm^{-1}): 1351 (m), 1332 (i), 1199 (i), 1137 (i), 1054 (l), 795 (m), 740 (m), 642 (m), 609 (m), 596 (m), 575 (m), 516 (m). ^{19}F NMR (1,4-dioxane-d_8; δ, ppm): -77.30 (s, CF_3), -77.31 (s, CF_3). ^{15}N NMR (1,4-dioxane-d_8; δ, ppm): 141.67 (s, $^{15}N\text{-}SO_2CF_3$). E.A. (calculated for $C_2AgF_6NO_4S_2$: C, 6.19; N, 3.61; S, 16.53) found: C, 6.17; N, 3.64; S, 16.33.

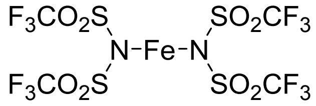

IR (ν, cm^{-1}): 1630 (l), 1352 (SO_2, m), 1332 (SO_2, i), 1199 (CF_3, vi), 1141 (SO_2, i), 1057 (S-N-S, m), 797 (C-S, m), 742 (CF_3, l), 645 (S-N-S, m), 597 (SO_2, m), 573 (CF_3, m), 517 (CF_3, m). ^{19}F NMR (1,4-dioxane-d_8; δ, ppm): -57.70 (s, 4xCF_3).

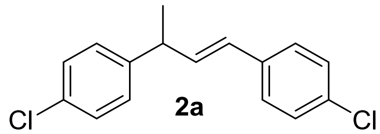

2a

The reaction crude was purified by preparative TLC on silica (run in *n*-hexane). Rf (*n*-hexane): 0.55. IR (ν, cm^{-1}): 3024 (d, =C-H), 2970 (mi, Csp3-H), 2870 (i), 1589 (vi, C=C), 1404 (l), 1092 (i), 1011 (m), 964 (m), 814 (i, C-Cl). GC/MS (m/z, $M^{+\cdot}$ 277), major peaks found: 276 (39%), 261 (57%), 241 (100%), 226 (43%), 191 (33%), 163 (14%), 149 (43%), 125 (26%), 101 (12%), 77 (9%). ^{1}H NMR (δ, ppm; *J*, Hz): 7.22-7.18 (m, 2H), 7.18-7.16 (m, 4H), 7.12-7.07 (m, 2H), 6.26 (d, *J* = 15.9, 1H), 6.19 (d, *J* = 15.8, 1H), 3.52 (qd, *J* = 7.0, 4.6, 1H), 1.35 (d,

J = 7.0, 3H). ^{13}C NMR (δ, ppm): 143.89 (C), 135.97 (C), 135.45 (2xCH), 132.90 (C), 132.14 (C), 128.79 (2xCH), 128.77 (2xCH), 127.89 (CH), 127.50 (3xCH), 42.09 (CH), 21.21 (CH_3).

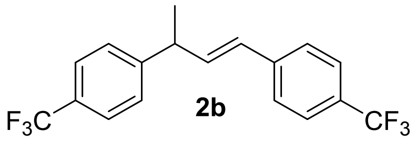

2b

The reaction crude was purified by preparative TLC on silica (run in *n*-hexane). Rf (*n*-hexane): 0.38. IR (ν, cm-1): 3055 (d, =C-H), 2985 (d, Csp3-H), 2920 (l), 1527 (l, C=C), 1323 (l), 1265 (i), 1122 (l), 744 (vi, CF_3). CG-MS (m/z, $M^{+\cdot}$ 344), major peaks found: 344 (39%), 329 (38%), 314 (4%), 275 (100%), 260 (14%), 197 (10%), 183 (56%), 172 (2%), 159 (31%), 129 (10%), 109 (6%). NMR ^{1}H (δ, ppm; J, Hz): 7.60 (d, J = 8.1, 2H), 7.55 (d, J = 8.2, 2H), 7.45 (d, J = 8.1, 2H), 7.39 (d, J = 8.4, 2H), 6.56-6.30 (m, 2H), 3.83-3.65 (m, 1H), 1.51 (d, J = 7.0, 3H). NMR ^{13}C (δ, ppm; J, Hz): 149.22 (C), 140.82 (C), 136.91 (CH), 128.81 (2xC, q, $J^2_{C\text{-}F}$ = 30.2), 128.29 (CH), 127.80 (2xCH), 126.49 (2xCH), 125.67 (quint, $J^3_{C\text{-}F}$ = 3.8, 4xCH), 124.33 (q, $J^1_{C\text{-}F}$ = 272.0, CF_3), 124.32 (q, $J^1_{C\text{-}F}$ = 272.0, CF_3), 42.65 (CH), 21.03 (CH_3).

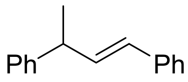

2c

The reaction crude was purified by preparative TLC on silica (run in *n*-hexane). Rf (*n*-hexane): 0.45. GC/MS (m/z, $M^{+\cdot}$ 208), major peaks found: 208 (100%), 193 (100%), 178 (71%), 165 (17%), 152 (9%), 130 (41%), 115 (100%), 103 (11%), 91 (57%), 77 (20%), 65 (11%), 51 (11%), 39 (8%). IR (ν, cm-1): 3062 (l, =C-H), 3024 (i, =C-H), 2962 (i, Csp^3-H), 2919 (l), 1597 (m, C=C), 1493 (i), 1450 (CH_3, i), 964 (i). ^{1}H NMR (δ, ppm; J, Hz): 7.52-7.25 (m, 10H), 6.52 (d, J = 15.3, 1H), 6.46 (d, J = 15.3, 1H), 3.74 (m, 1H), 1.57 (d, J = 7.0, 3H). ^{13}C NMR (δ, ppm): 145.6 (C), 137.5 (C), 135.2 (CH), 128.5 (CH), 128.4 (4xCH), 127.3 (2xCH), 127.0 (CH), 126.2 (CH), 126.1 (2xCH), 42.5 (CH), 21.2 (CH_3).

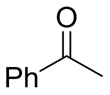

4a

The reaction crude was purified by preparative TLC on silica (run in *n*-hexane/AcOEt mixtures). GC-MS (m/z, $M^{+\cdot}$ 120), major peaks found: 120 (40%), 105 (100%), 77 (40%), 51 (40%). ^{1}H NMR (δ, ppm; *J*, Hz): 7.94-7.88 (m, 2H), 7.55-7.47 (m, 1H), 7.45-7.37 (m, 2H), 2.56 (s, 3H).

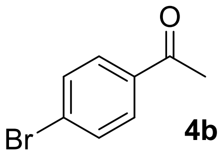

4b

The reaction crude was purified by preparative TLC on silica (run in *n*-hexane/AcOEt mixtures). GC-MS (m/z, $M^{+\cdot}$ 198), major peaks found: 200 (95%), 198 (95%), 185 (100%), 183 (100%), 157 (100%), 155 (100%), 76 (45%), 50 (35%). ^{1}H NMR (δ, ppm; *J*, Hz): 7.92 (dt, *J* = 9.0, 2.5, 2H), 6.91 (dt, *J* = 9.0, 2.5, 2H), 2.56 (s, 3H).

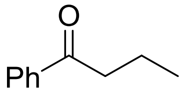

4c

The reaction crude was purified by preparative TLC on silica (run in *n*-hexane/AcOEt mixtures). GC-MS (m/z, $M^{+\cdot}$ 148), major peaks found: 148 (33%), 120 (20%), 105 (100%), 77 (92%), 51 (20%). ^{1}H NMR (δ, ppm; *J*, Hz): 7.96 (d, *J* = 7.2, 2H), 7.55 (t, *J* = 7.2, 1H), 7.45 (t, *J* = 7.2, 2H), 2.94 (t, *J* = 7.3, 2H), 1.78 (sext, *J* = 7.3, 2H), 1.00 (t, *J* = 7.3, 3H).

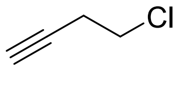

The reaction crude was purified by distillation.[S3-S4] IR (ν, cm^{-1}): 3335 (i, Csp3-H), 3324 (i), 3005 (l, C-H), 2967 (l, C-H), 2924 (l, C-H), 2358 (l), 2339 (m), 1275 (i), 1260 (i), 764 (vi), 751 (vi), 645 (vi). GC-MS (m/z, $M^{+\cdot}$ 88), major peaks found: 88 (50 %), 73 (1 %), 62 (2 %), 53 (100 %), 39 (6 %). ^{1}H NMR (δ, ppm; *J*, Hz): 3.60 (t, *J* = 7.2, 2H), 2.66 (td, *J* = 7.2, 2.6, 2H), 2.08 (t, *J* = 2.6, 1H). ^{13}C NMR (δ, ppm): 80.35 (C), 70.55 (CH), 42.04 (CH_2-Cl), 23.00 (CH_2).

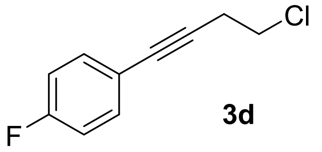

3d

The reaction crude was purified by column chromatography using as eluent (*n*-hexane). R_f (*n*-hexane): 0.50. IR (ν, cm^{-1}): 2959 (l, C-H), 2920 (l, C-H), 2000-1600 (l, overtones), 1508 (vi), 1232 (i), 1157 (l), 834 (i), 533 (m). GC-MS (m/z, $M^{+\cdot}$ 182), major peaks found: 182 (50 %), 146 (25 %), 133 (100 %), 120 (12 %), 107 (10 %). ^{1}H NMR (δ, ppm; *J*, Hz): 7.47-7.31 (m, 2H), 7.03-6.94 (m, 2H), 3.67 (t, *J* = 7.2, 2H), 2.86 (t, *J* = 7.2, 2H). ^{13}C NMR (δ, ppm; *J*, Hz): 162.50 (d, $J^1_{C\text{-}F}$ = 249.1, C), 133.65 (d, $J^3_{C\text{-}F}$ = 8.3, 2xCH), 119.33 (d, $J^4_{C\text{-}F}$ = 3.5, C), 115.63 (d, $J^2_{C\text{-}F}$ = 22.0, 2xCH), 85.48 (d, $J^5_{C\text{-}F}$ = 1.5, C), 81.55 (C), 42.26 (CH_2), 23.89 (CH_2).

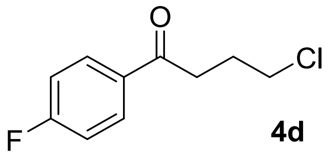

4d

The reaction crude was purified by column chromatography using as eluent (20 % AcOEt in *n*-hexane). R_f (20 % AcOEt in *n*-hexane): 0.66. GC-MS (m/z, $M^{+\cdot}$ 200), major peaks found: 200 (1 %), 164 (1 %), 138 (43 %), 123 (100 %), 107 (10 %). ^{1}H NMR (δ, ppm; *J*, Hz): 8.07-7.94 (m, 2H), 7.20-7.07 (m, 2H), 3.68 (t, *J* = 6.2, 2H), 3.15 (t, *J* = 7.0, 2H), 2.22 (quint, *J* = 6.7, 2H). ^{13}C NMR (δ, ppm; *J*, Hz): 197.48 (C=O), 165.97 (d, $J^1_{C\text{-}F}$ = 254.9, C), 133.35 (d, $J^4_{C\text{-}F}$ = 3.0, C), 130.80 (d, $J^3_{C\text{-}F}$ = 9.3, 2xCH), 115.90 (d, $J^2_{C\text{-}F}$ = 21.9, 2xCH), 44.76 (CH_2), 35.34 (CH_2), 26.86 (CH_2).

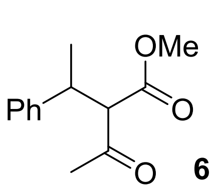

6

GC-MS (m/z, $M^{+\cdot}$ 220), major peaks found: 220 (2 %), 202 (100 %), 189 (8 %), 177 (29 %), 159 (21 %), 145 (97 %), 131 (40 %), 117 (18 %), 117 (83 %), 91 (13 %), 77 (17 %).

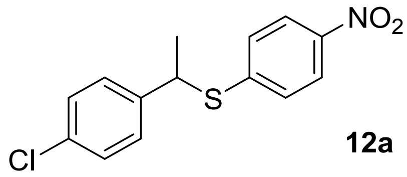

12a

The reaction crude was purified by preparative TLC on silica (run in 10 % Et_2O in *n*-hexane). R_f (10 % Et_2O in *n*-hexane): 0.41. IR (ν, cm^{-1}): 3098 (l, arC-H), 3066 (l, arC-H), 2971 (l, C-H), 2925 (l, C-H), 2866 (l, C-H), 2359 (l), 2337 (l), 2000-1600 (l, overtones), 1511 (i, arC-NO_2), 1492 (m, arC-C), 1477 (l, arC-C), 1447 (l, -CH_3), 1092 (i, arC-Cl), 820 (m), 681 (l, C-S). GC-MS (m/z, $M^{+\cdot}$ 293), major peaks found: 293 (4%), 278 (1%), 231 (2%), 155 (1%), 139 (100%), 103 (77 %), 77 (25 %). ^1H NMR (δ, ppm; *J*, Hz): 7.99-7.94 (m, 2H), 7.26-7.17 (m, 6H), 4.45 (q, *J* = 7.0, 1H), 1.58 (d, *J* = 7.0, 3H). ^{13}C NMR (δ, ppm): 145.82 (C), 145.77 (C), 140.79 (C), 133.52 (C), 129.10 (2xCH), 128.89 (2xCH), 128.56 (2xCH), 123.95 (2xCH), 46.01 (CH), 22.92 (CH_3).

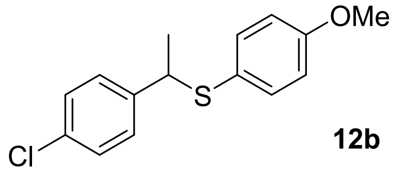

12b

The reaction crude was purified by preparative TLC on silica (run in 5 % AcOEt in *n*-hexane). R_f (5 % AcOEt in *n*-hexane): 0.58. IR (ν, cm^{-1}): 3064 (l, arC-H), 3023 (l, arC-H), 2961 (i, C-H), 2926 (i, C-H), 2864 (l, C-H), 2835 (m, C-H), 2000-1600 (l, overtones), 1490 (vi, arC-C), 1478 (i, arC-C), 1440 (i, -CH_3), 1246 (vi, arC-O-Me), 1092 (vi, arC-Cl), 827 (vi), 641 (m, C-S). GC-MS (m/z, $M^{+\cdot}$ 278), major peaks found: 278 (79%), 264 (2%), 140 (100%), 125 (29%), 103 (100%), 77 (43%). ^1H NMR (δ, ppm; *J*, Hz): 7.24-7.16 (m, 4H), 7.15-7.09 (m, 2H), 6.79-6.73 (m, 2H), 4.13 (q, *J* = 7.0, 1H), 3.78 (s, 3H), 1.57 (d, *J* = 7.0, 3H). ^{13}C NMR (δ, ppm): 159.86 (C), 142.16 (C), 136.27 (2xCH), 132.66 (C), 128.80 (2xCH), 128.48 (2xCH), 124.67 (C), 114.39 (2xCH), 55.37 (O-CH_3), 48.57 (CH), 21.79 (CH_3). HRMS (ESI) [M+H^+; calculated for $C_{15}H_{16}ClOS$: 279.0611] found *m/z* 279.0632.

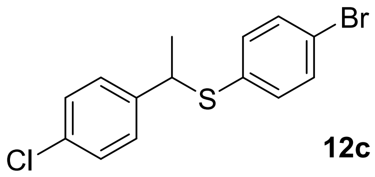

12c

The reaction crude was purified by preparative TLC on silica (run in *n*-hexane). R_f (*n*-hexane): 0.45. IR (ν, cm^{-1}): 3045 (l, arC-H), 3026 (l, arC-H), 2965 (m, C-H), 2924 (l, C-H), 2865 (l, C-H), 2000-1600 (l, overtones), 1490 (m, arC-C), 1472 (m, arC-C), 1445 (l, $-CH_3$), 1091 (vi, arC-Cl), 1068 (m, arC-Br), 861 (l), 815 (br), 688 (l, C-S). GC-MS (m/z, $M^{+\cdot}$ 328), major peaks found: 328 (43%), 313 (4%), 207 (5%), 189 (16%), 139 (100%), 103 (100%), 77 (57%). ^{1}H NMR (δ, ppm; *J*, Hz): 7.37-7.31 (m, 2H), 7.26-7.21 (m, 2H), 7.21-7.16 (m, 2H), 7.13-7.07 (m, 2H), 4.26 (q, J = 7.0, 1H), 1.59 (d, J = 7.1, 3H). ^{13}C NMR (δ, ppm): 141.62 (C), 134.39 (2xCH), 133.81 (C), 133.04 (C), 132.01 (2xCH), 128.73 (2xCH), 128.71 (2xCH), 121.83 (C), 47.65 (CH), 22.27 (CH_3).

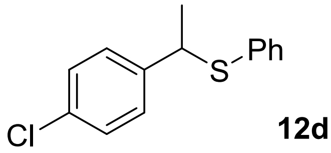

12d

The reaction crude was purified by preparative TLC on silica (run in *n*-hexane). R_f (*n*-hexane): 0.48. IR (ν, cm^{-1}): 3073 (m, arC-H), 3058 (m, arC-H), 2968 (m, C-H), 2924 (m, C-H), 2864 (m, C-H), 2000-1600 (l, overtones), 1490 (vi, arC-C), 1479 (m, arC-C), 1439 (l, $-CH_3$), 1093 (vi, arC-Cl), 828 (vi), 791 (m), 690 (m, C-S). GC-MS (m/z, $M^{+\cdot}$ 248), major peaks found: 248 (46%), 233 (2%), 197 (4%), 141 (100%), 103 (100%), 77 (46%). ^{1}H NMR (δ, ppm; *J*, Hz): 7.36-7.26 (m, 2H), 7.25-7.18 (m, 7H), 4.31 (q, *J* = 7.1, 1H), 1.61 (d, J = 7.0, 3H). ^{13}C NMR (δ, ppm): 142.01 (C), 134.69 (C), 132.88 (2xCH), 129.20 (C), 128.90 (2xCH), 128.74 (2xCH), 128.62 (2xCH), 127.53 (CH), 47.58 (CH), 22.35 (CH_3).

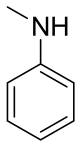

14a

The reaction crude was purified by preparative TLC on silica (run in *n*-hexane:AcOEt 4:1). GC/MS (m/z, $M^{+\cdot}$ 107), major peaks found: 106 (100 %), 77 (21 %), 51 (7 %). ^{1}H NMR (300 MHz, $CDCl_3$) δ 7.34 – 7.16 (m, H), 6.77 (t, *J* = 7.3 Hz, 2H), 6.71 – 6.59 (m, 1H), 3.43 (s, 1H), 2.88 (s, 3H).

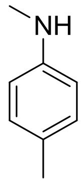

14b

The reaction crude was purified by preparative TLC on silica (run in *n*-hexane:AcOEt 4:1). GC/MS (m/z, $M^{+\cdot}$ 121), major peaks found: 120 (100 %), 91 (16 %), 65 (6 %). ^{1}H NMR (300 MHz, $CDCl_3$) δ 7.04 (d, J = 8.5 Hz, 2H), 6.53 (d, J = 7.9 Hz, 2H), 4.32 (s, 1H), 2.75 (s, 3H), 2.18 (s, 3H).

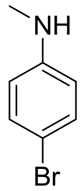

14c

The reaction crude was purified by preparative TLC on silica (run in *n*-hexane:AcOEt 4:1). GC/MS (m/z, $M^{+\cdot}$ 185), major peaks found: 185 (100 %), 104 (25 %), 77 (20 %). ^{1}H NMR (300 MHz, $CDCl_3$) δ 7.16 (d, J = 2.0 Hz, 2H), 6.51 (d, J = 3.6 Hz, 2H), 3.65 (s, 1H), 2.80 (s, 3H).

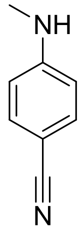

14d

The reaction crude was purified by preparative TLC on silica (run in *n*-hexane:AcOEt 4:1). GC/MS (m/z, $M^{+\cdot}$ 132), major peaks found: 131 (100 %), 102 (10 %), 75 (7 %). ^{1}H NMR (300 MHz, $CDCl_3$) δ 7.37 (dd, J = 9.1, 2.2 Hz, 2H), 6.52 (d, J = 8.8 Hz, 2H), 3.37 (s, 1H), 2.81 (s, 3H).

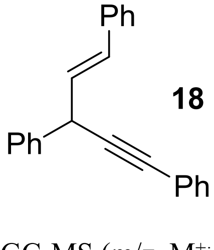

18

GC-MS (m/z, $M^{+\cdot}$ 294), major peaks found: 294 (100 %), 279 (24 %), 215 (85 %), 202 (31 %), 189 (36 %), 165 (10 %), 115 (10 %), 91 (12 %).

Figures.

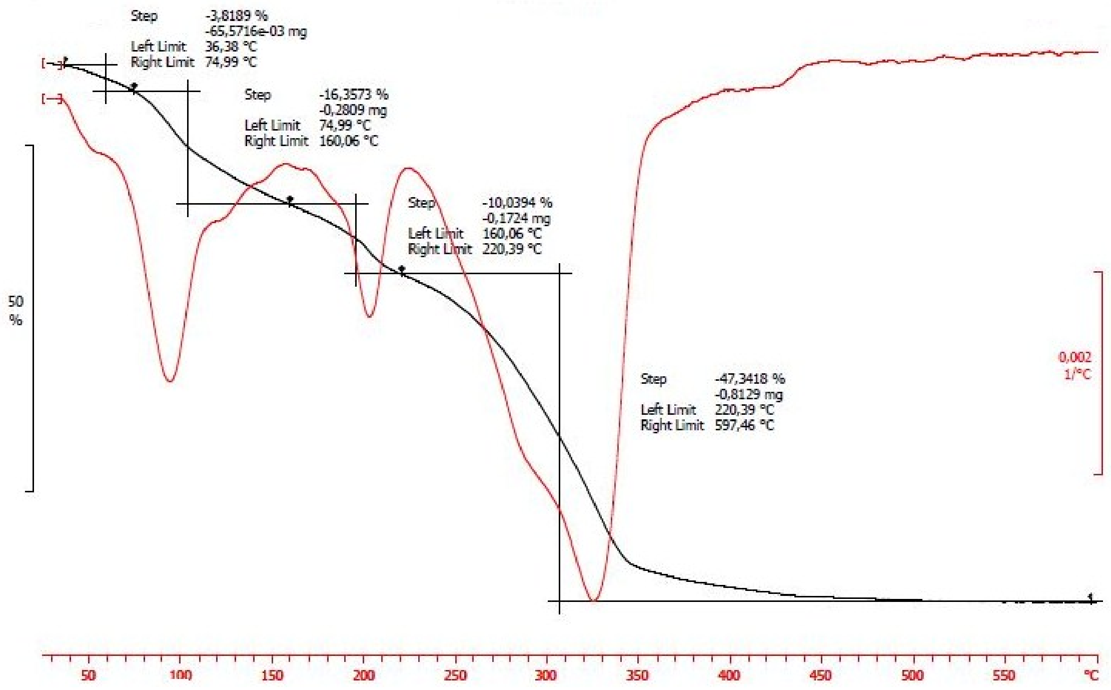

Figure S1. Thermogravimmetric (TG) analysis of $Fe_2O(NTf_2)_5$@AgNPs.

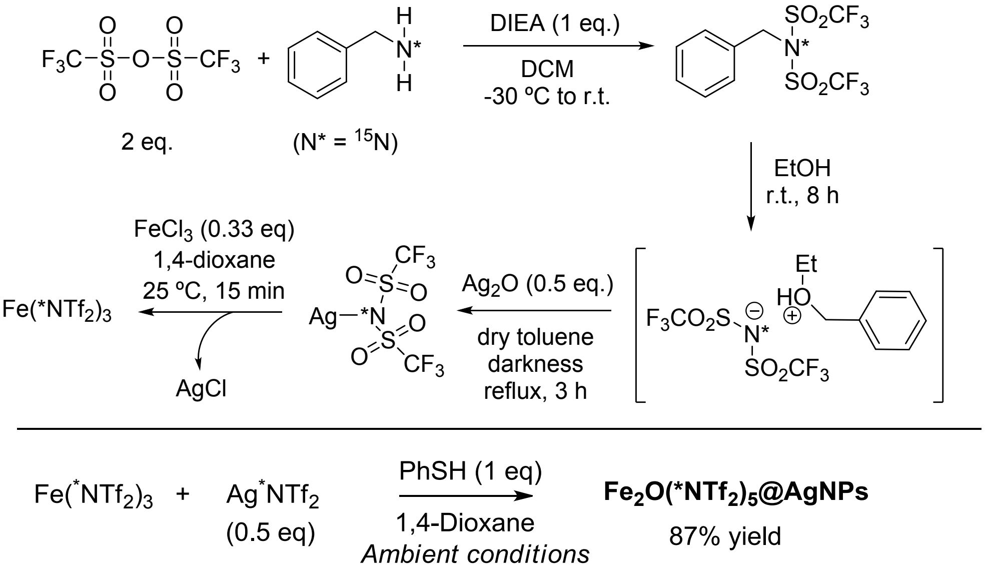

Figure S2. Preparation of isotopically labeled *15N*-$Fe_2O(NTf_2)_5$@AgNPs using *15N*-silver(I) and *15N*-iron(III) triflimide as starting materials. The isotopically labeled ^{15}N-silver(I) triflimide was synthetized from *15N*-benzylamine.

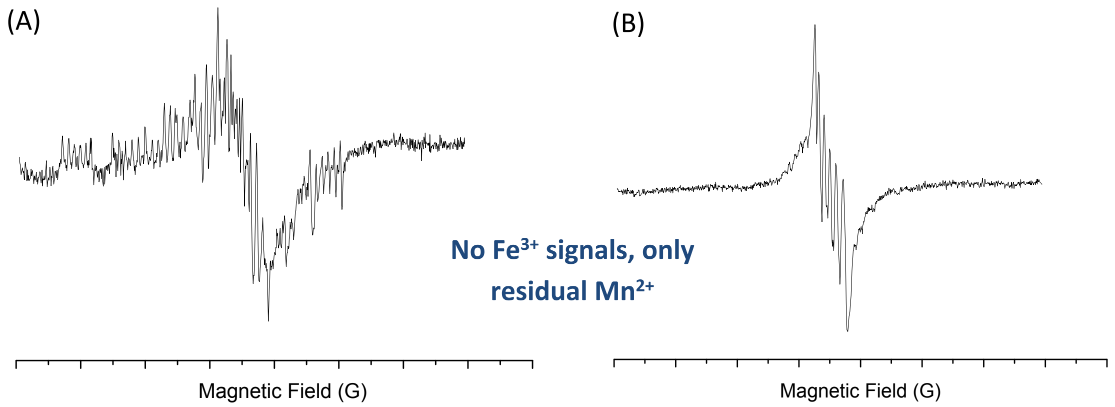

Figure S3. Electronic Paramagnetic Resonance (EPR) spectra of $Fe_2O(NTf_2)_5$@AgNPs (Ag:Fe ratio= 0.5) (A) before and (B) after evacuation at 70 ºC during 30 min. The minor signal observed corresponds to residual Mn^{2+} in the starting Fe source.

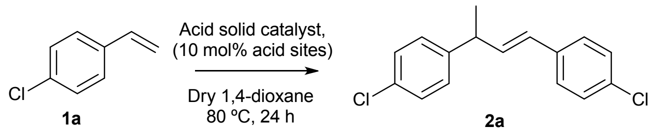

Catalyst	Initial rate (h^{-1})	Final yield (%)
$Fe_2O(NTf_2)_5$@AgNPs	7.9	98
Nafion^{TM}	23.0	99
H-USY zeolite	2.8	43
Sulfated zirconia	2.5	31

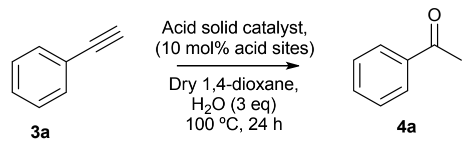

Catalyst	Initial rate (h^{-1})	Final yield (%)
$Fe_2O(NTf_2)_5$@AgNPs	15.0	97
Nafion^{TM}	22.5	99
H-USY zeolite	7.0	74
Sulfated zirconia	2.8	29

Figure S4. Comparison of the catalytic results of $Fe_2O(NTf_2)_5$@AgNPs, nafion^{TM}, H-USY zeolite and sulfated zirconia for the acid-catalysed dimerization of styrenes (top) and hydration of alkynes (bottom). GC yields.

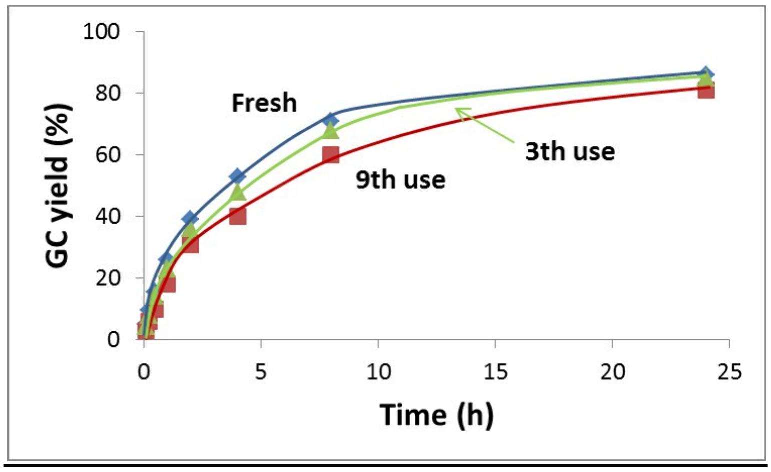

Figure S5. Kinetic results for the hydrothiolation of 4–chlorostyrene **1a** with thiophenol catalyzed by fresh and reused $Fe_2O(NTf_2)_5$@AgNPs catalyst.

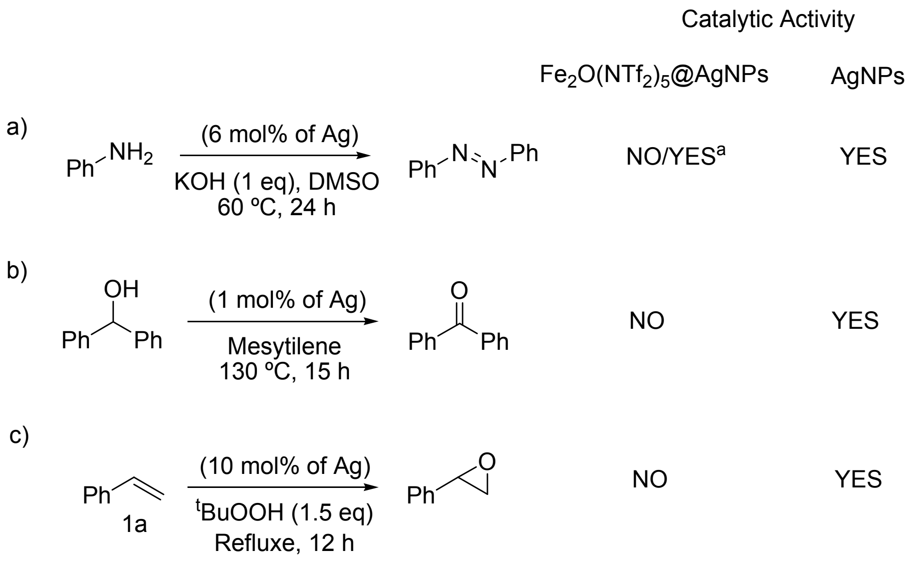

Figure S6. Reactions selected for the accessibility study of reagents to Ag NPs in the $Fe_2O(NTf_2)_5$@AgNPs solid: a) synthesis of azobenzene from aniline, b) aerobic dehydrogenation of diphenylmethanol and c) epoxidation of styrene **1a**. [a] With exposed Ag NPs on surface (se main text).

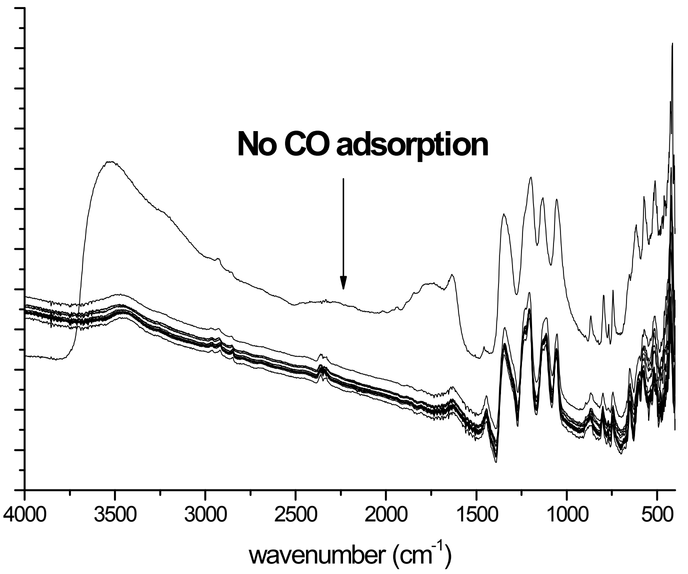

Figure S7. Low-temperature FT-IR spectrum of $Fe_2O(NTf_2)_5$@AgNPs (Ag:Fe ratio= 0.5) with carbon monoxide (CO) as a probe molecule.

Tables.

Table S1. FT-IR bands for $Fe_2O(NTf_2)_5$@AgNPs (Ag:Fe molar ratio= 0.5).

Vibration	Frequency (cm^{-1})	Vibration	Frequency (cm^{-1})
$\nu_{as}(SO_2)$	1351, 1331	$\delta s(CF_3)$	742, 731
$\nu_s(CF_3)$	1248, 1241	$\delta(SNS)$	646, 642
$\nu_{as}(CF_3)$	1203, 1197	$\delta(SO_2)$	609, 597
$\nu_s(SO_2)$	1146, 1141, 1138	$\delta a(CF_3)$	574, 515
$\nu_a(SNS)$	1067, 1055	ν_{as}(Fe-O-Fe)	867
$\nu(CS)$	796	Unassigned Bands	1630, 686, 695
$\nu_s(SNS)$	771		

The more relevant bands corresponds to the triflimide vibrations which by comparison with previous FT-IR studies on different triflimides could be assigned. The band situated at 1055 cm^{-1} corresponds to ν_a(SNS), indicating that the triflimides in the solid are deprotonated, since the partial double bond character acquired due to the electron delocalization shifts the band dramatically to higher frequencies with respect to the acid form (860 cm^{-1}). To determine whether the anion is coordinated to the Fe^{3+} metal center or not, $\nu_s(SO_2)$ and $\nu_{as}(SO_2)$ are the most sensitive vibrations. While $\nu_s(SO_2)$ shows no significant shift with respect to the triflimidic acid, $\nu_{as}(SO_2)$ does show an appreciable change and, moreover, this band has two peaks that could indicate different coordination modes for the anion. Based on these data, one must conclude that the triflimide anions are coordinated to the metal ion through the sulfonyl groups in, at least, two different ways.

Although of lower intensity, a band at 865 cm^{-1} reveals the presence of μ-hidroxo bridges. This frequency is normally expected for monobridged structures, decreasing to 760-780 cm^{-1} for dibridged and 720-750 cm^{-1} for tribridged compounds. According to reported correlations between the angle of the Fe-O-Fe entity and the ν_{as}(Fe-O-Fe) stretch, the high frequency observed in our case indicates that the angle is closer to 180^0. These data point to the presence of a single μ-oxo or μ-hidroxo bridge with another bridging triflimide, which would explain the different SO_2 bands observed.

Table S2. Comparison of the state-of-the-art catalysts for the reaction studied with the triflimide solid catalysts under similar reaction conditions.

Reaction	State-of-the-art catalysts	Ref.	This work (triflimide solid)
Head–to–tail dimerization of styrenes	Noble metal complexes (mainly Pd). No solid catalysts found.	9-14	Same yields than soluble catalysts (up to 98%)
Markovnikov hydration of alkynes	Noble metal salts and complexes (mainly Au, but also Pd, Co, Fe,…), soluble Brönsted acids under harsh conditions and particular solid acids in high wt% loadings	15-21	Same yields (up to 97%)
Addition of methyl acetoacetate to styrene	Pd and Fe salts and complexes. No solid catalyst found.	22-24	90% yield.
Hydrodeoxygenation of cyclohexanol	Metal triflates-Pt/C (150 ºC), Pt-TiO_2/C (300 ºC)	25-27	98% yield.
Selective –CH_2– oxidation with H_2O_2	Fe complexes	31-35	moderate conversions and selectivity
Baeyer-Villiger oxidation	Metal complexes and solids	36	moderate conversions and selectivity
Markovnikov hydrothiolation of styrenes	Fe^{3+} triflimide	37-38	Same yields than soluble catalysts (up to 88%)
Vinylation of 1,3–diphenylpropargyl alcohol	Noble metal, Fe and Cu salts. Fe triflate-impregnated in particular triflimide modified charcoal	46-48	Similar yield than soluble catalyst (68%)
Demethylation of N,N-dimethyanilines	Fe complexes with strong oxidants. For the aerobic process, addition of metal triflates with yields <40%.	39-40	Similar yields than the Fe complex/metal triflate combined catalyst
One pot hydration of alkynes–CH2 oxidation	-	41	Low yield, but regioselective

Schemes

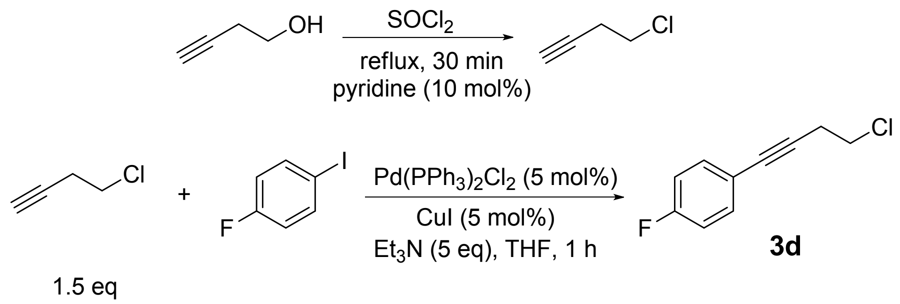

Scheme S1. Two-step preparation of the starting material compound **3d.** Step 1: Chlorination of homopropargylic alcohol with thionyl chloride in the presence of catalytic amounts of pyridine. Step 2: Sonogashira cross-coupling between homopropargylic chloride and *p*-iodofluorobenzene.

NMR spectra

^{1}H-NMR

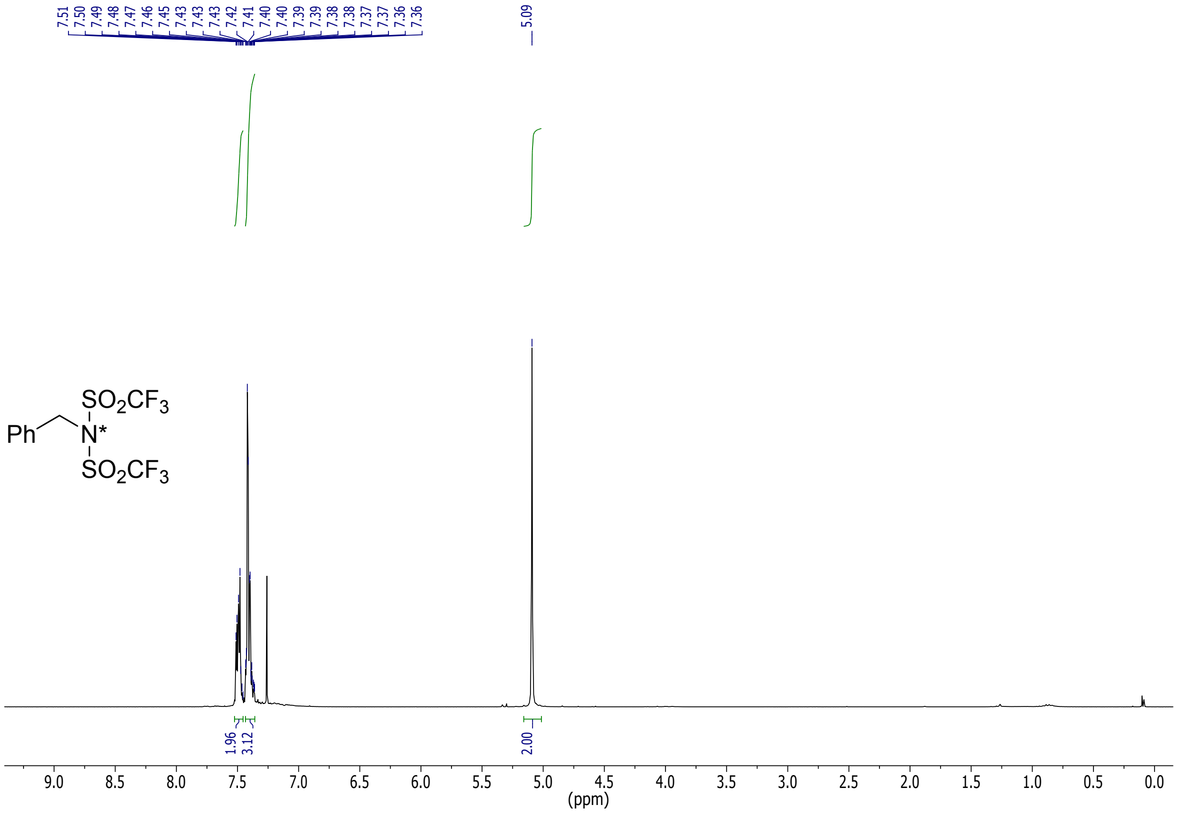

^{13}C-NMR

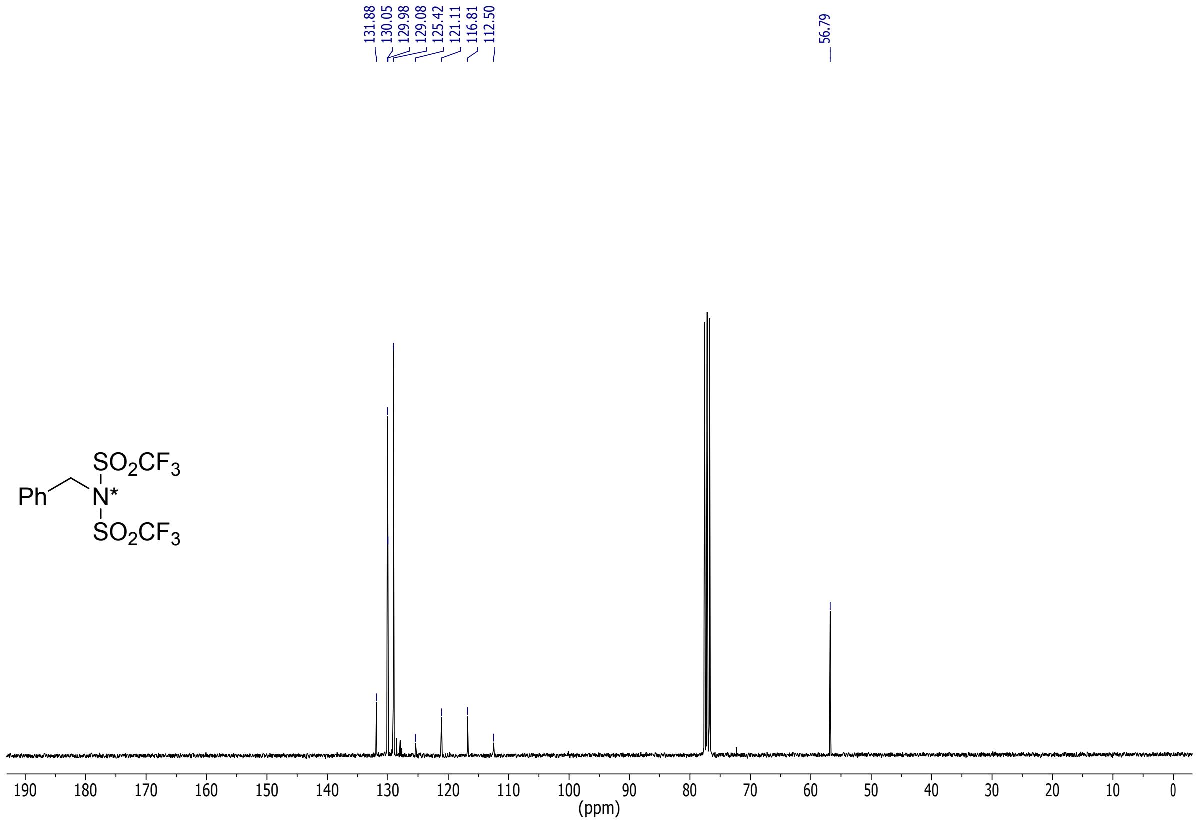

^{19}F-NMR

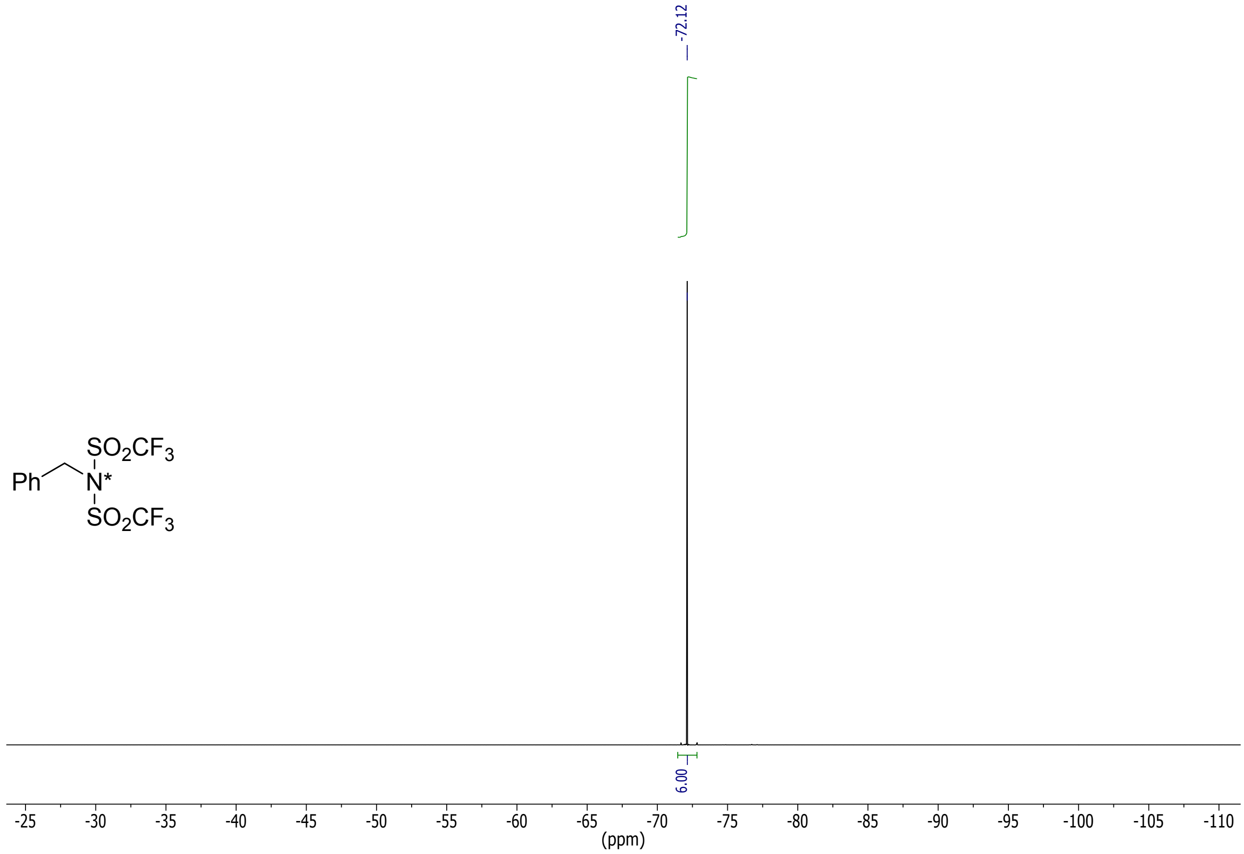

^{19}F-NMR

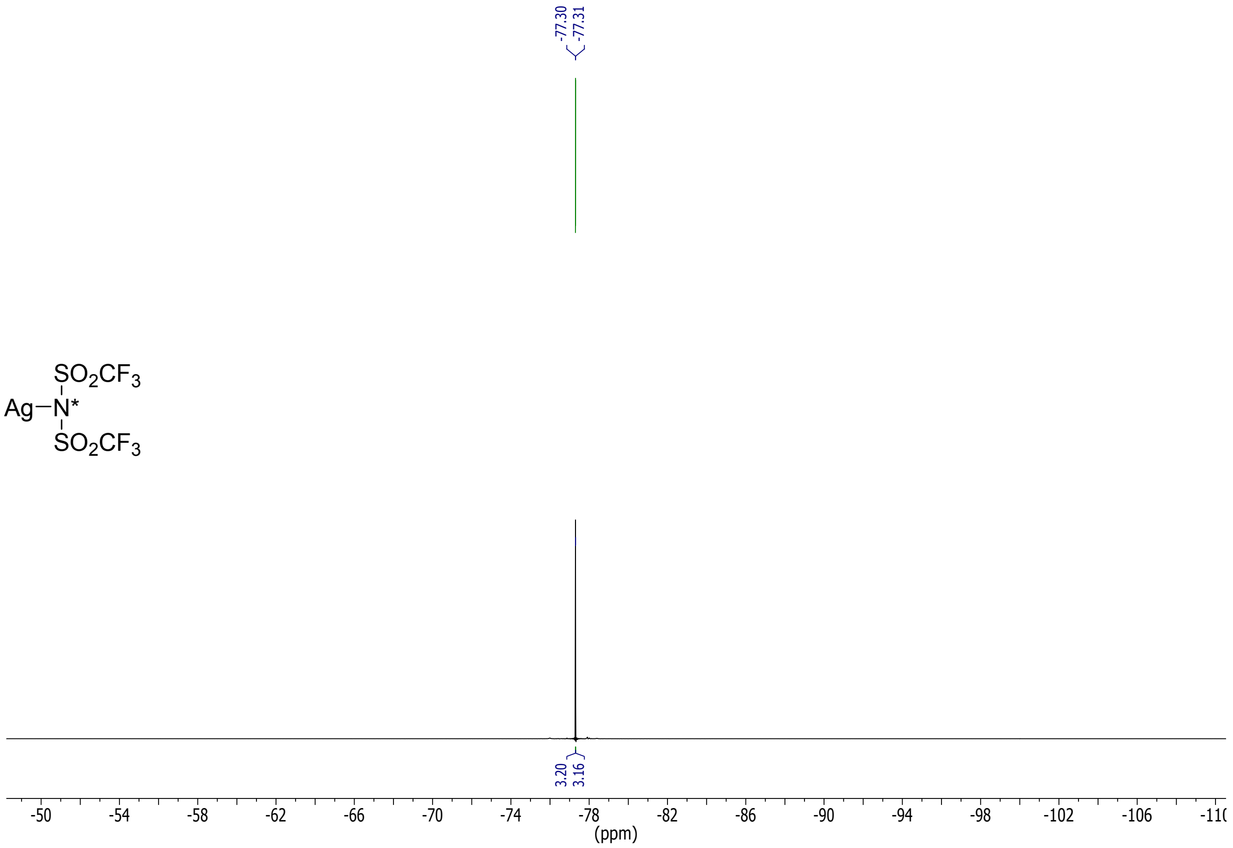

^{15}N-NMR

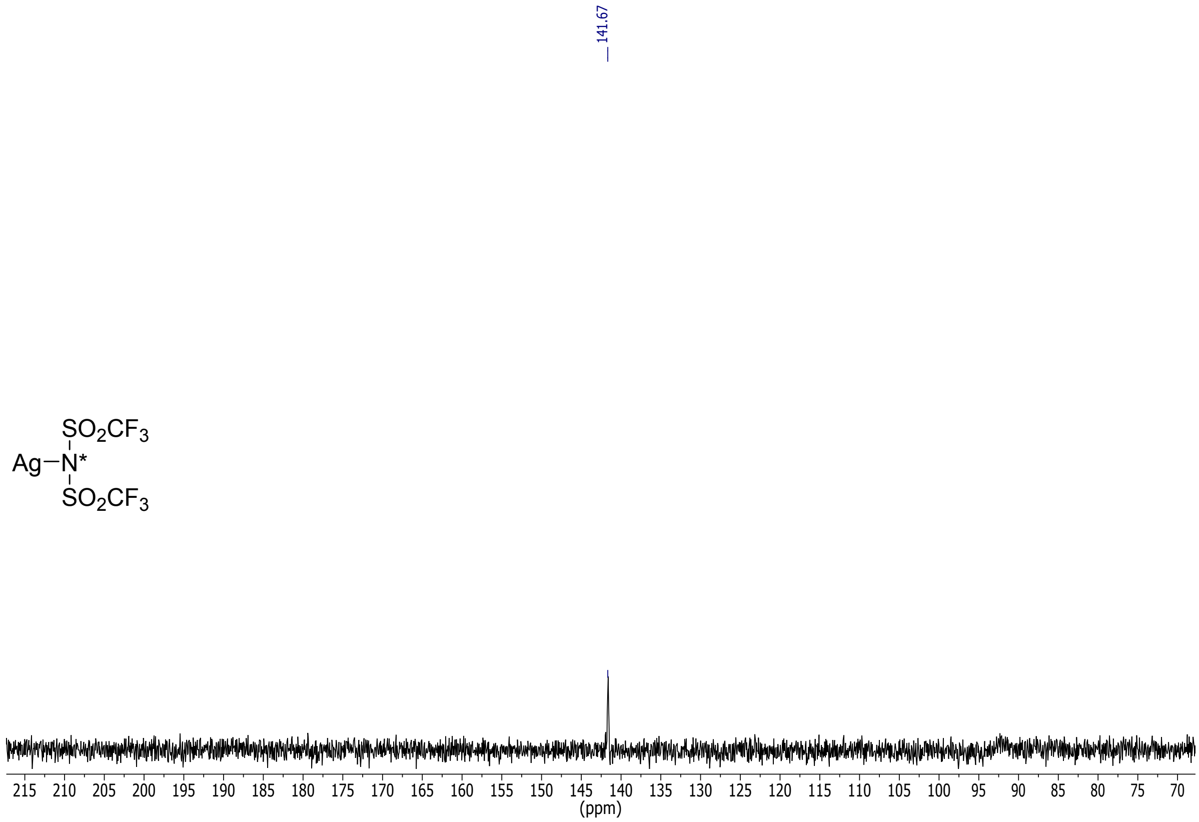

^{1}H-NMR

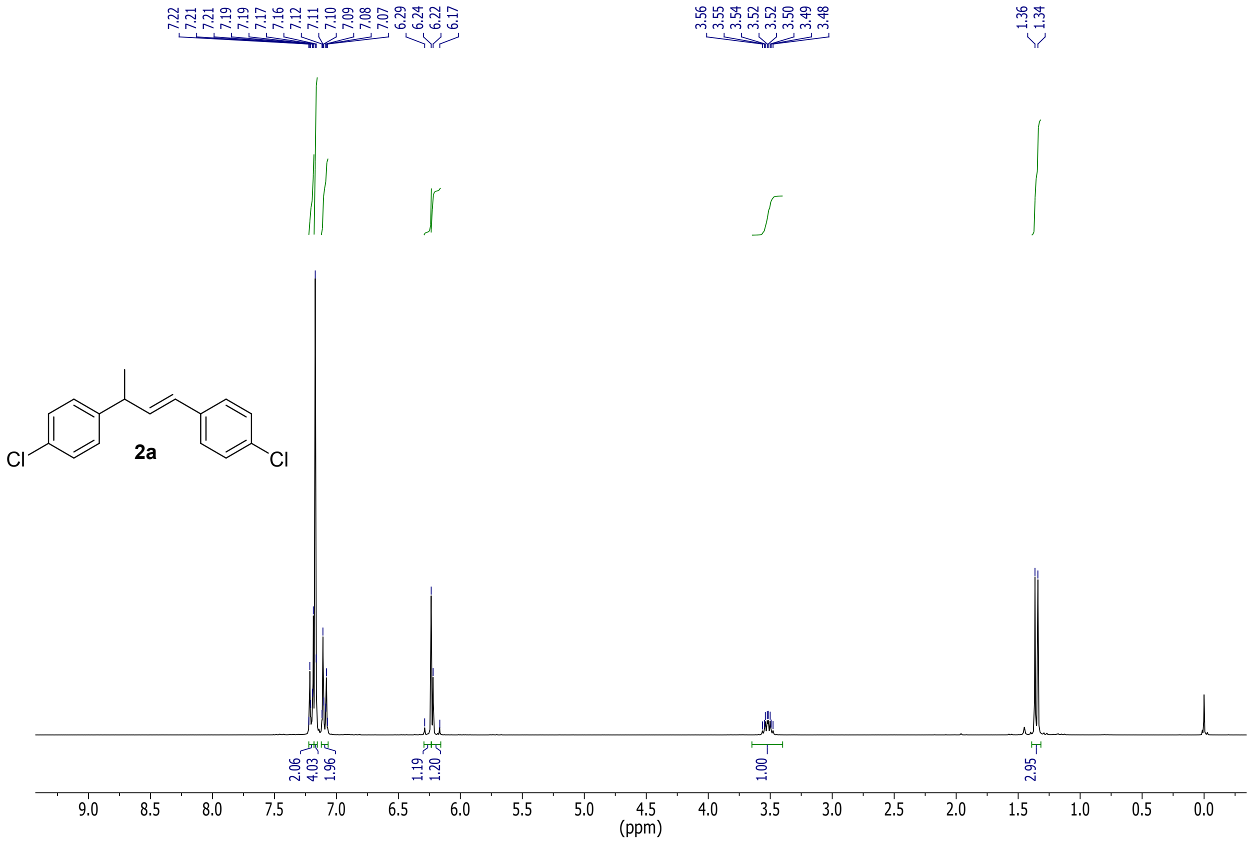

^{13}C-NMR

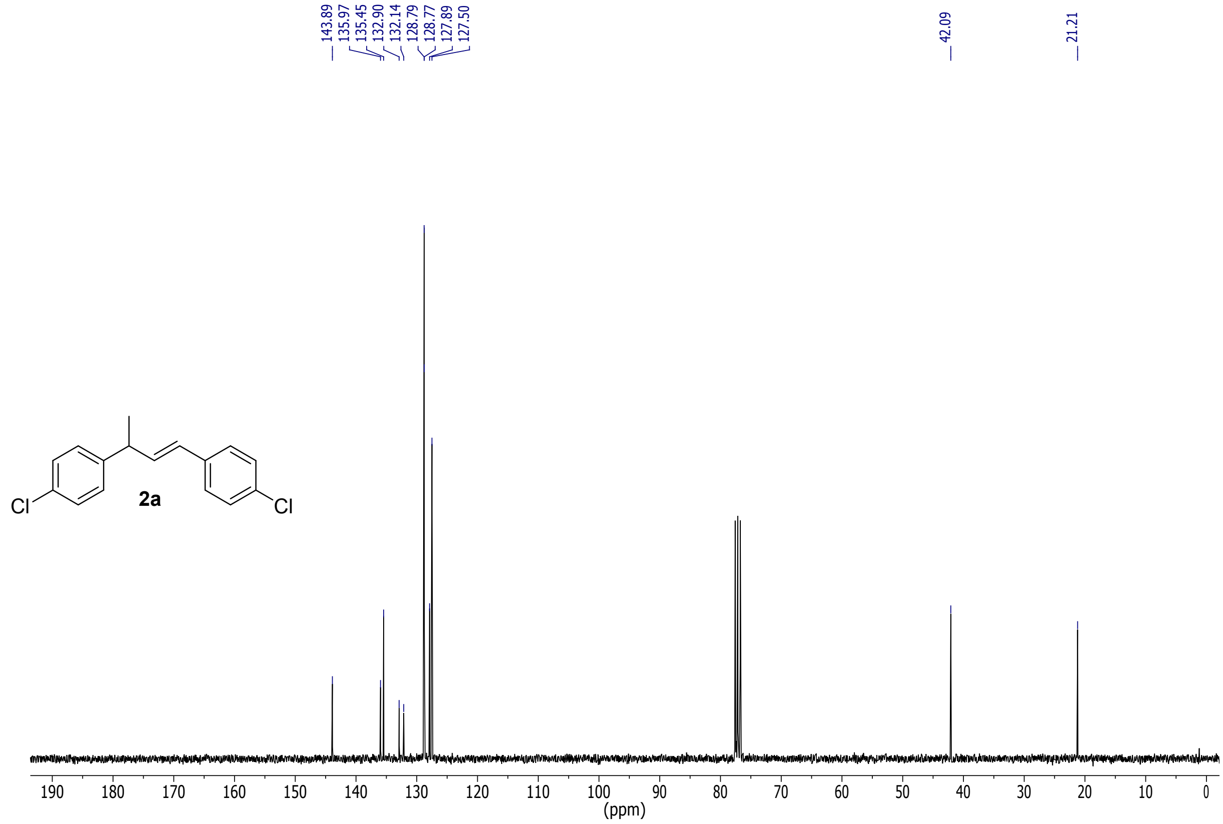

^{1}H-NMR

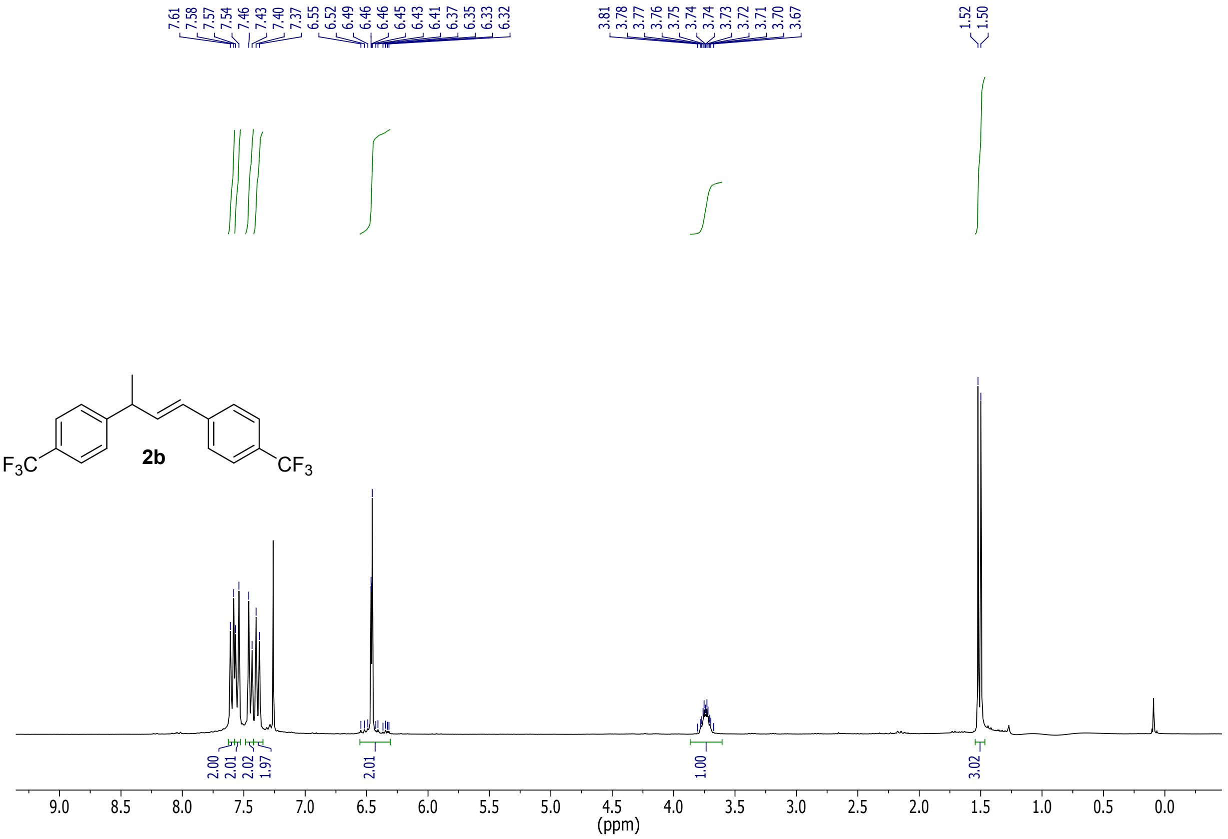

^{13}C-NMR

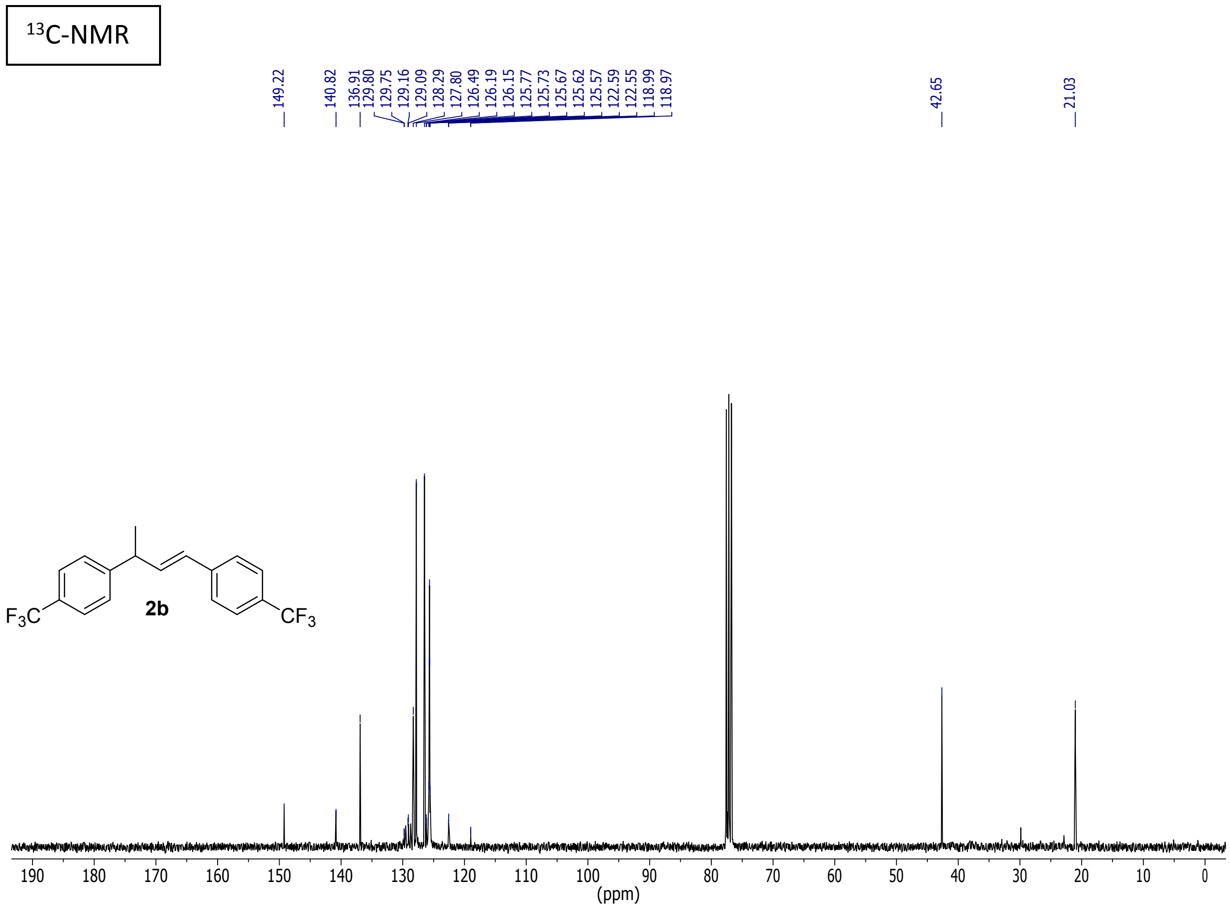

^{1}H-NMR

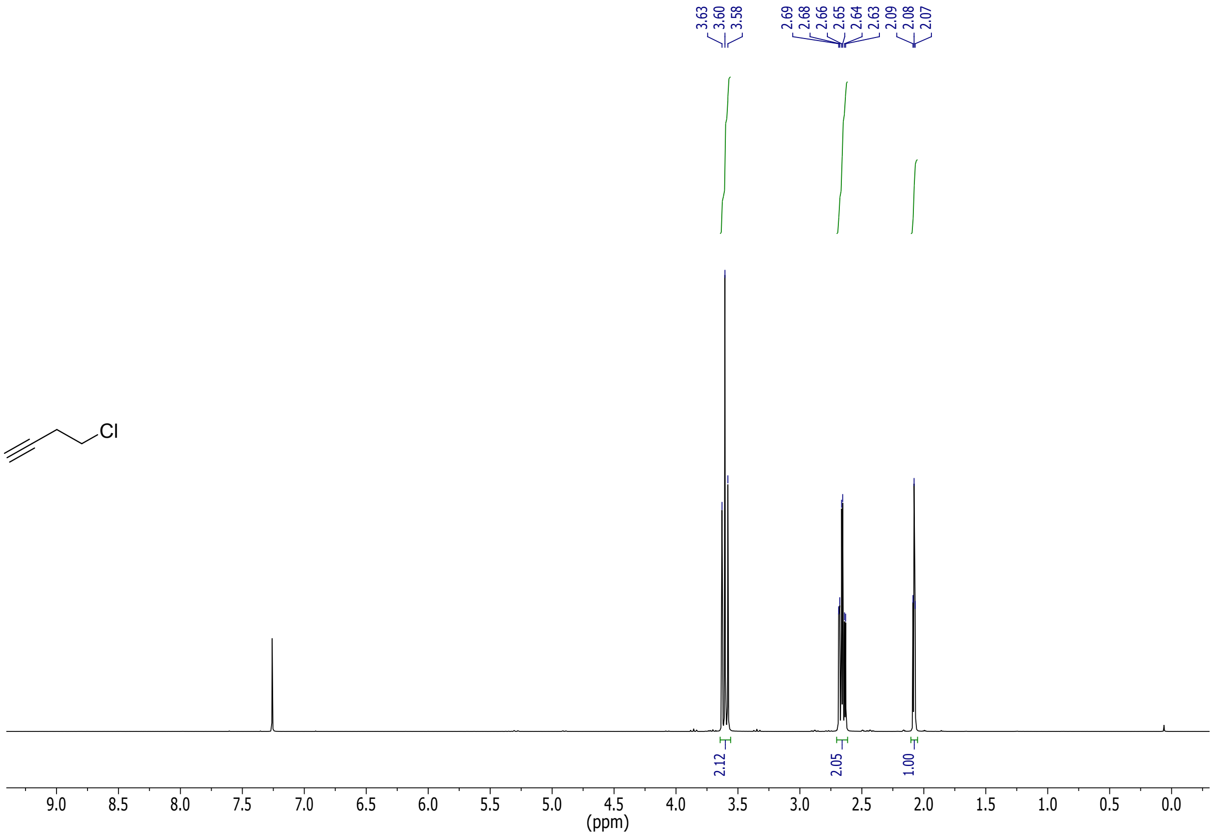

^{13}C-NMR

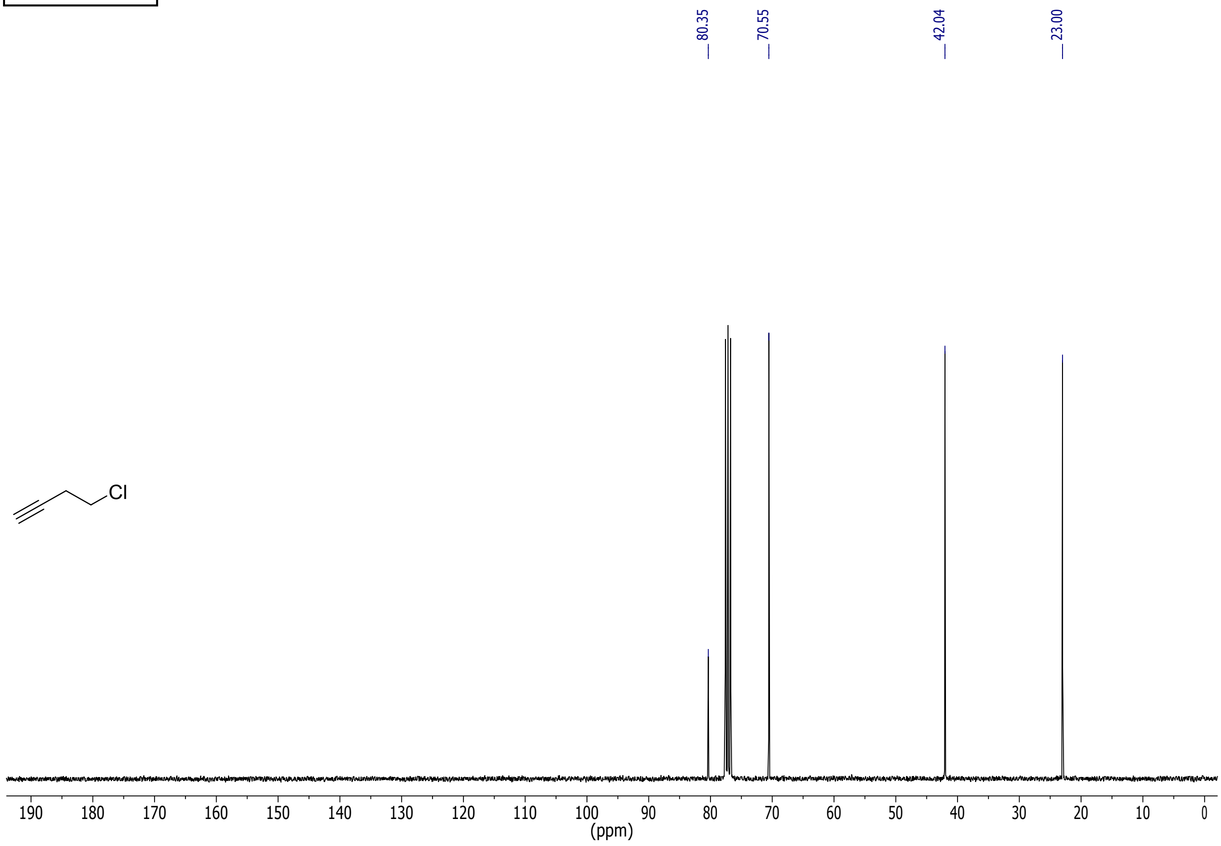

^{1}H-NMR

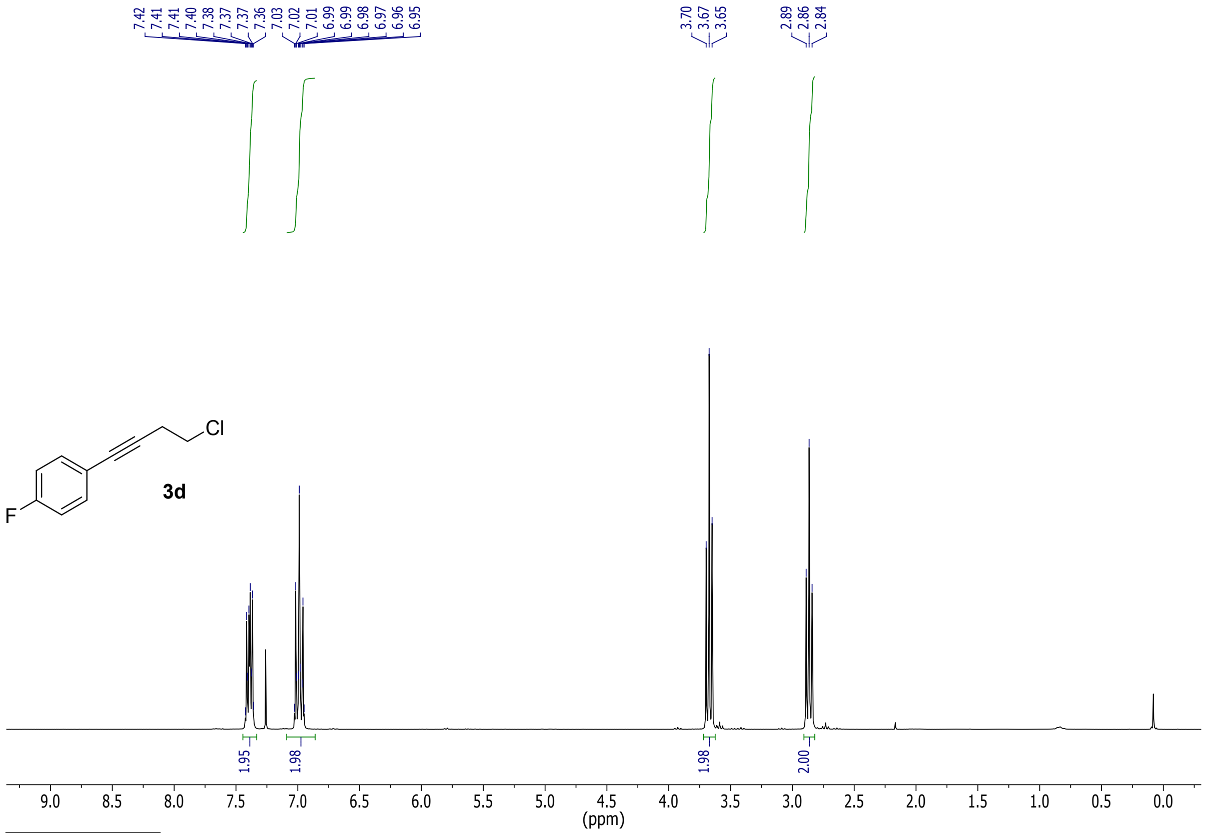

^{13}C-NMR

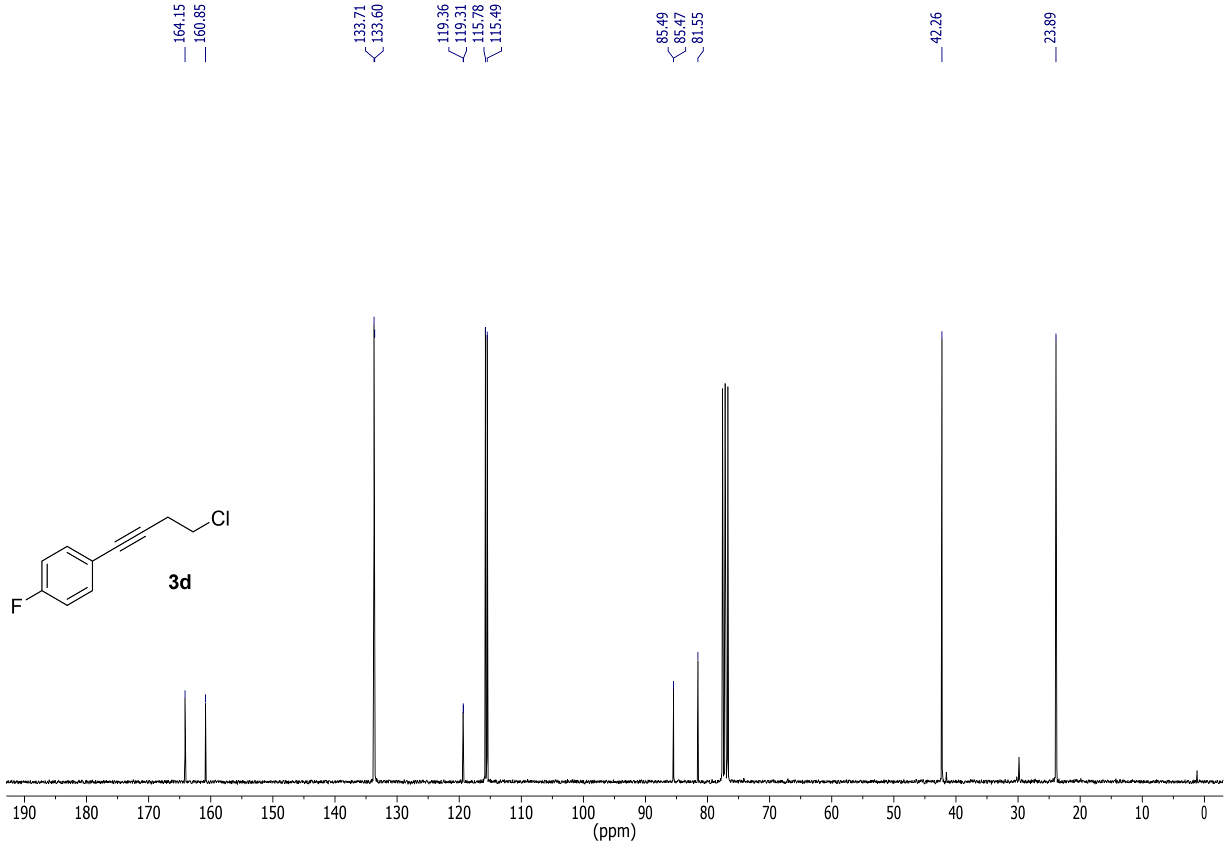

^{1}H-NMR

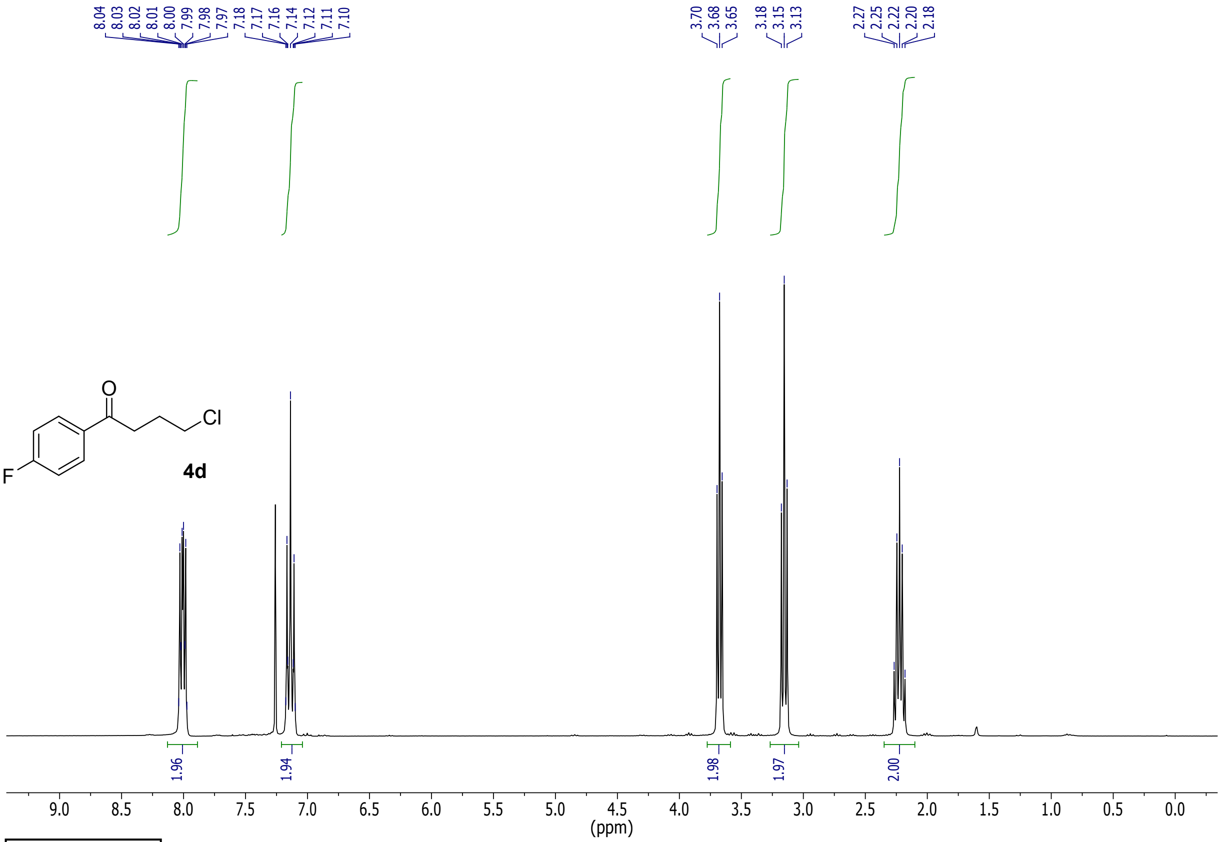

^{13}C-NMR

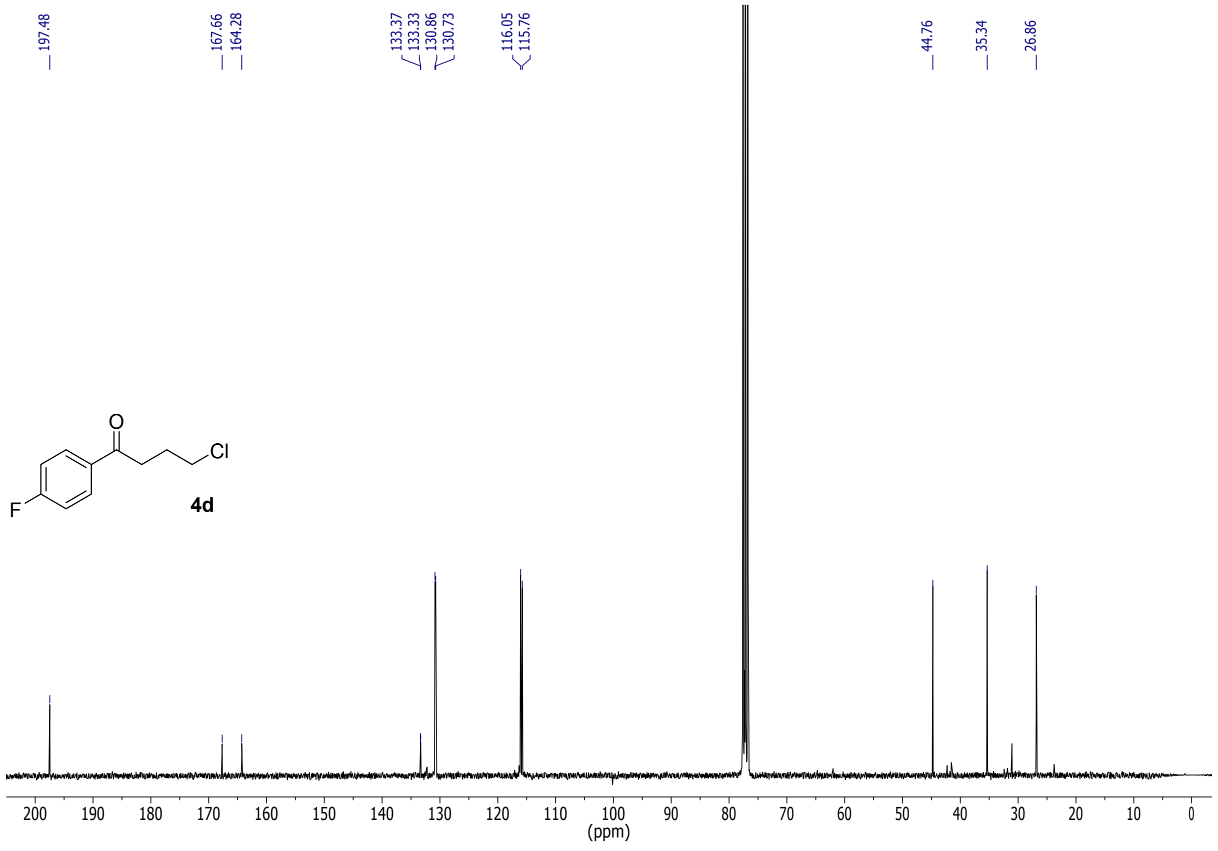

^{1}H-NMR

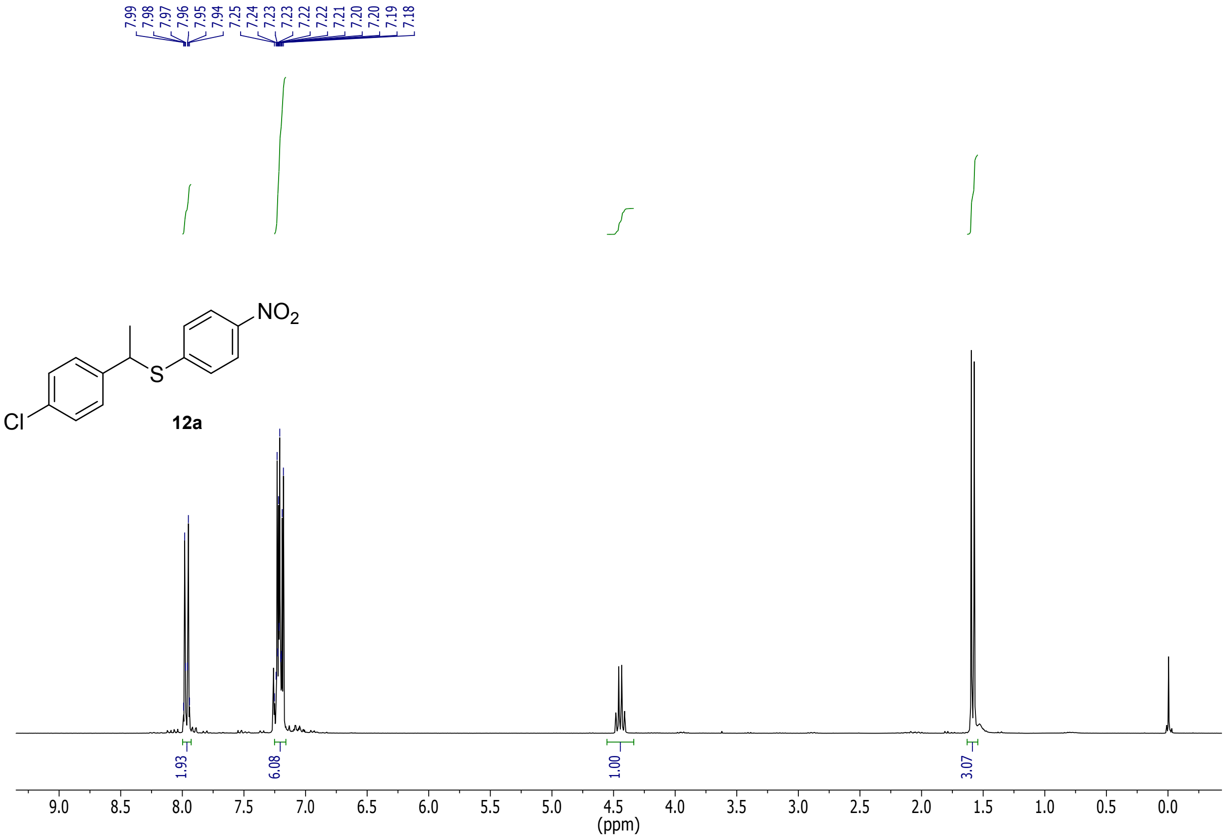

^{13}C-NMR

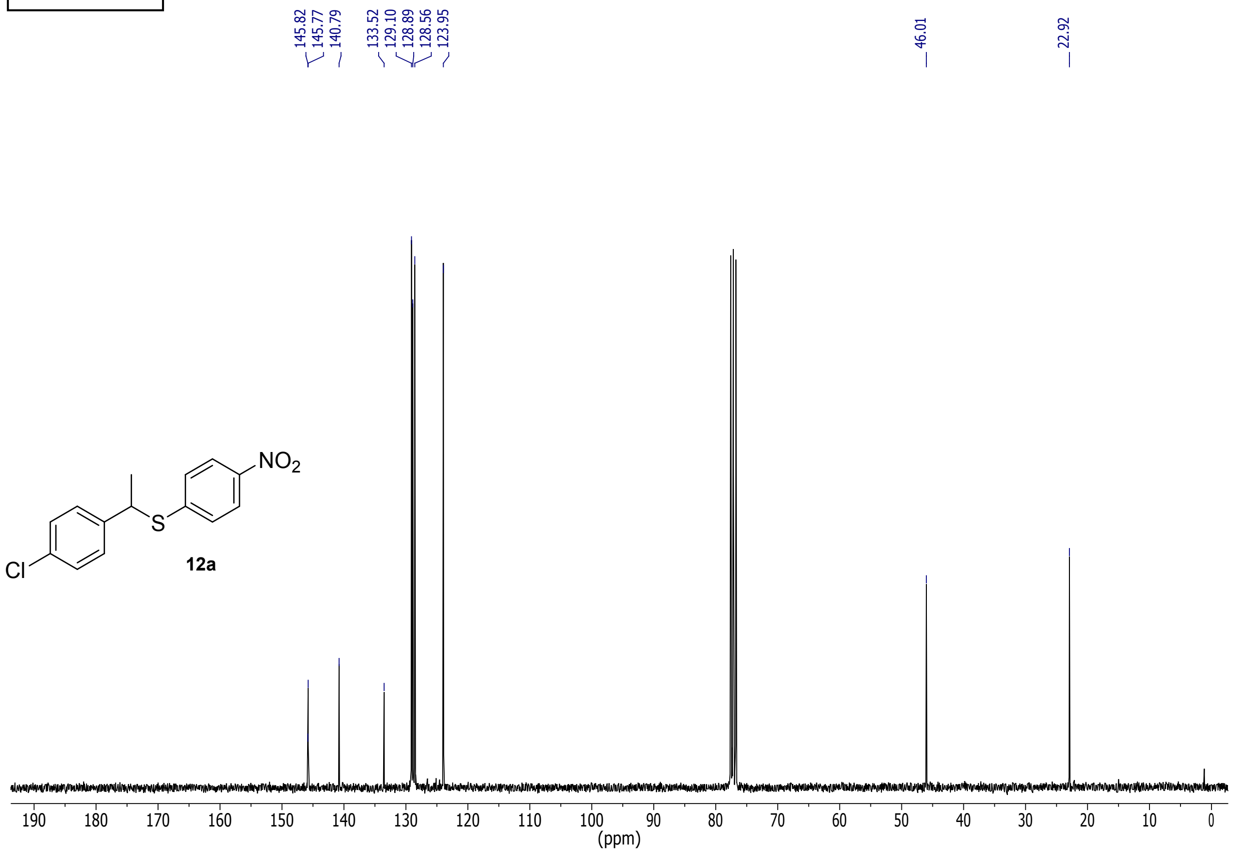

^{1}H-NMR

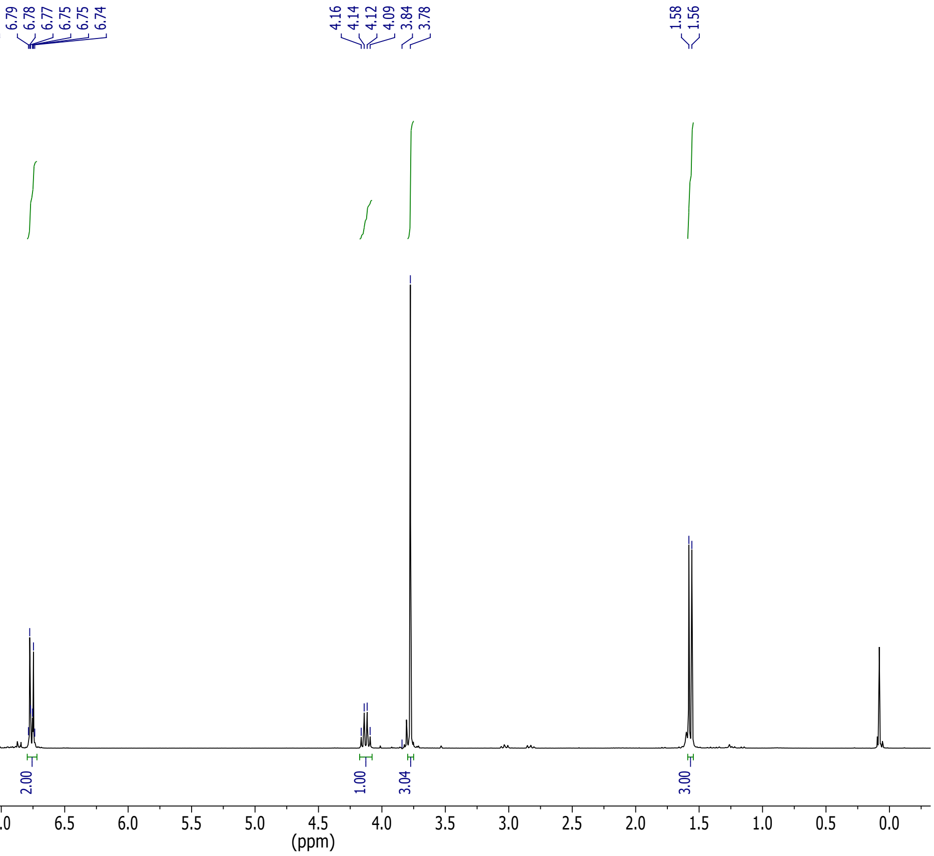

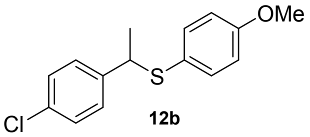

OMe
S
Cl
12b

13C-NMR

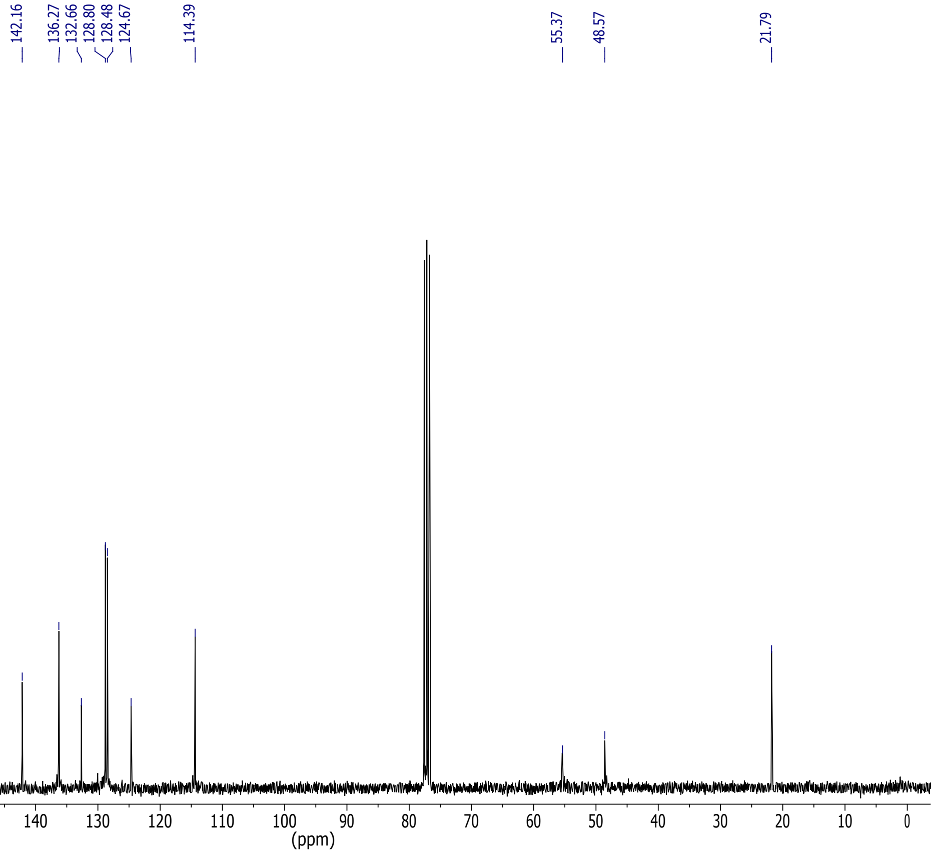

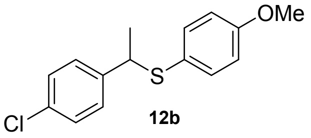

OMe
S
Cl
12b

^{1}H-NMR

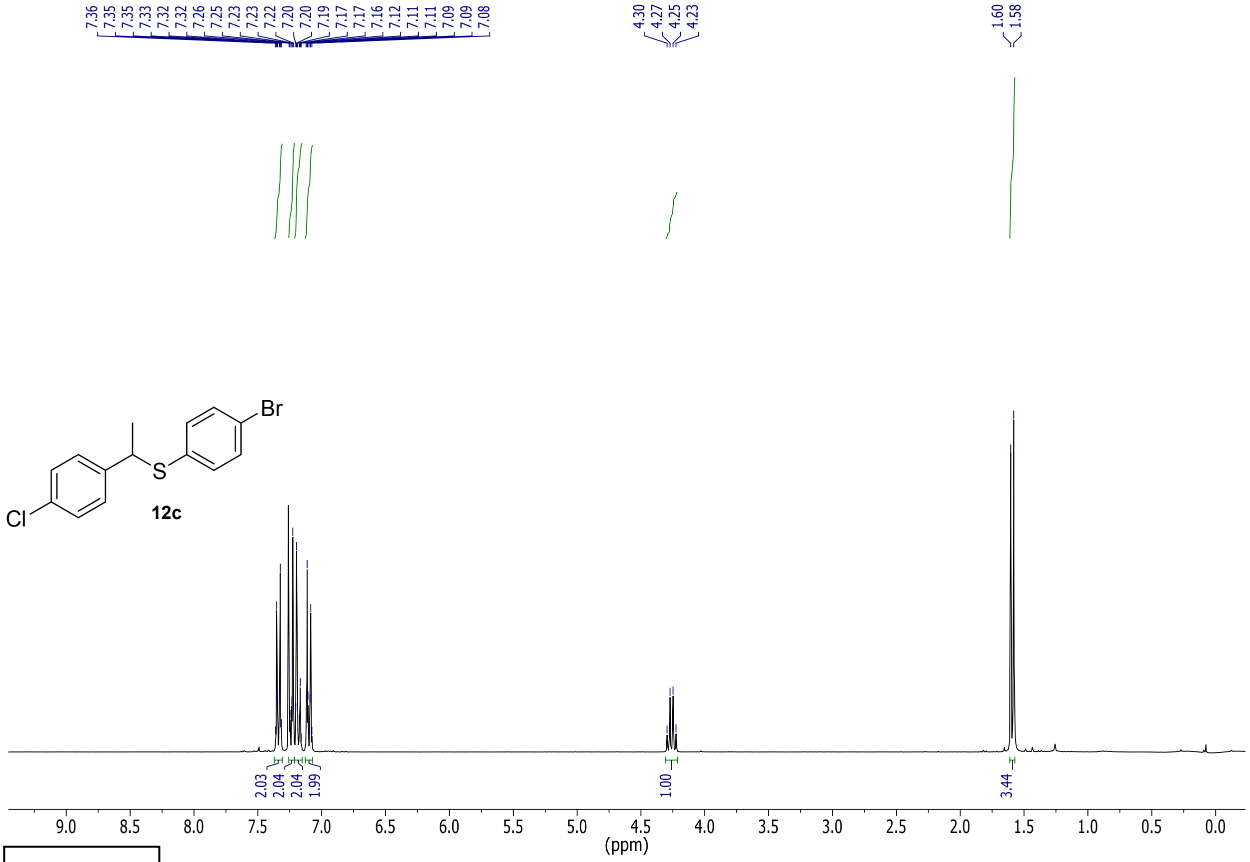

^{13}C-NMR

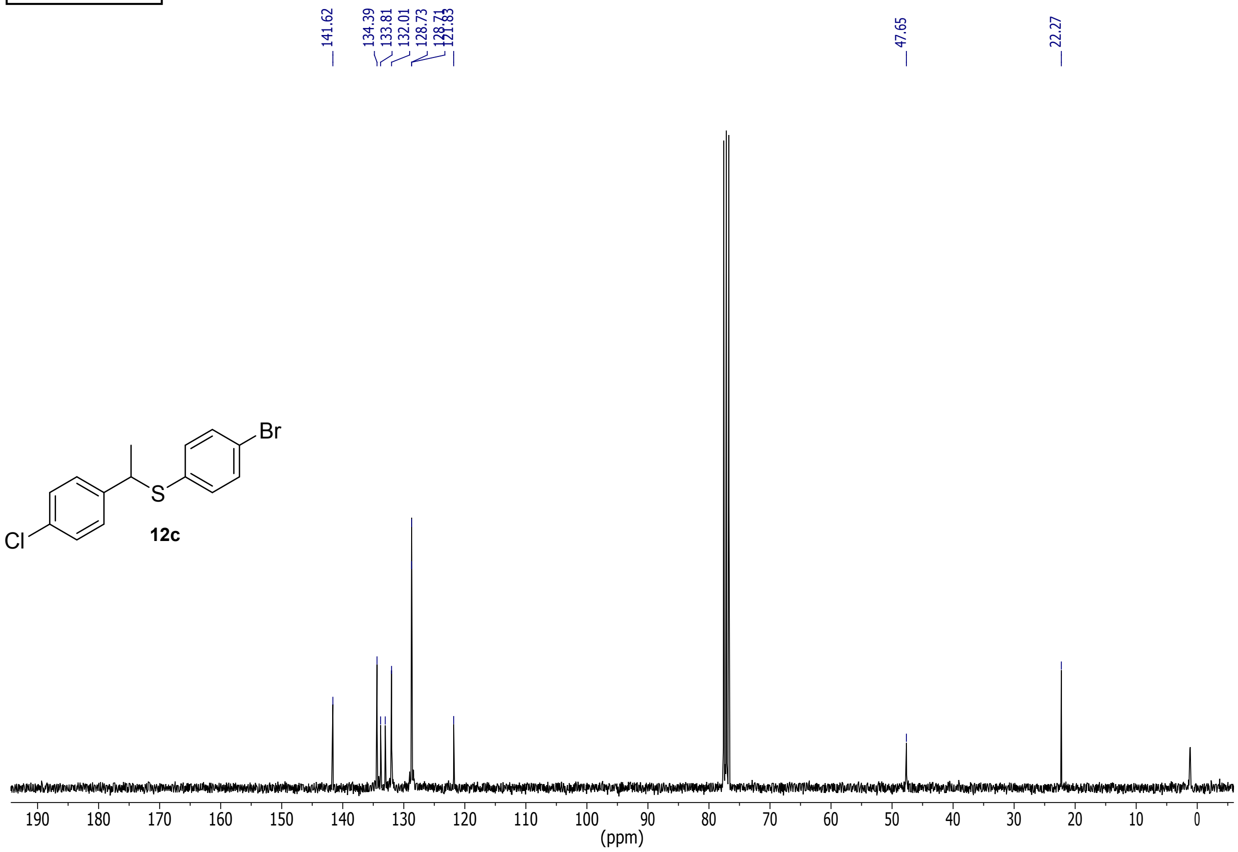

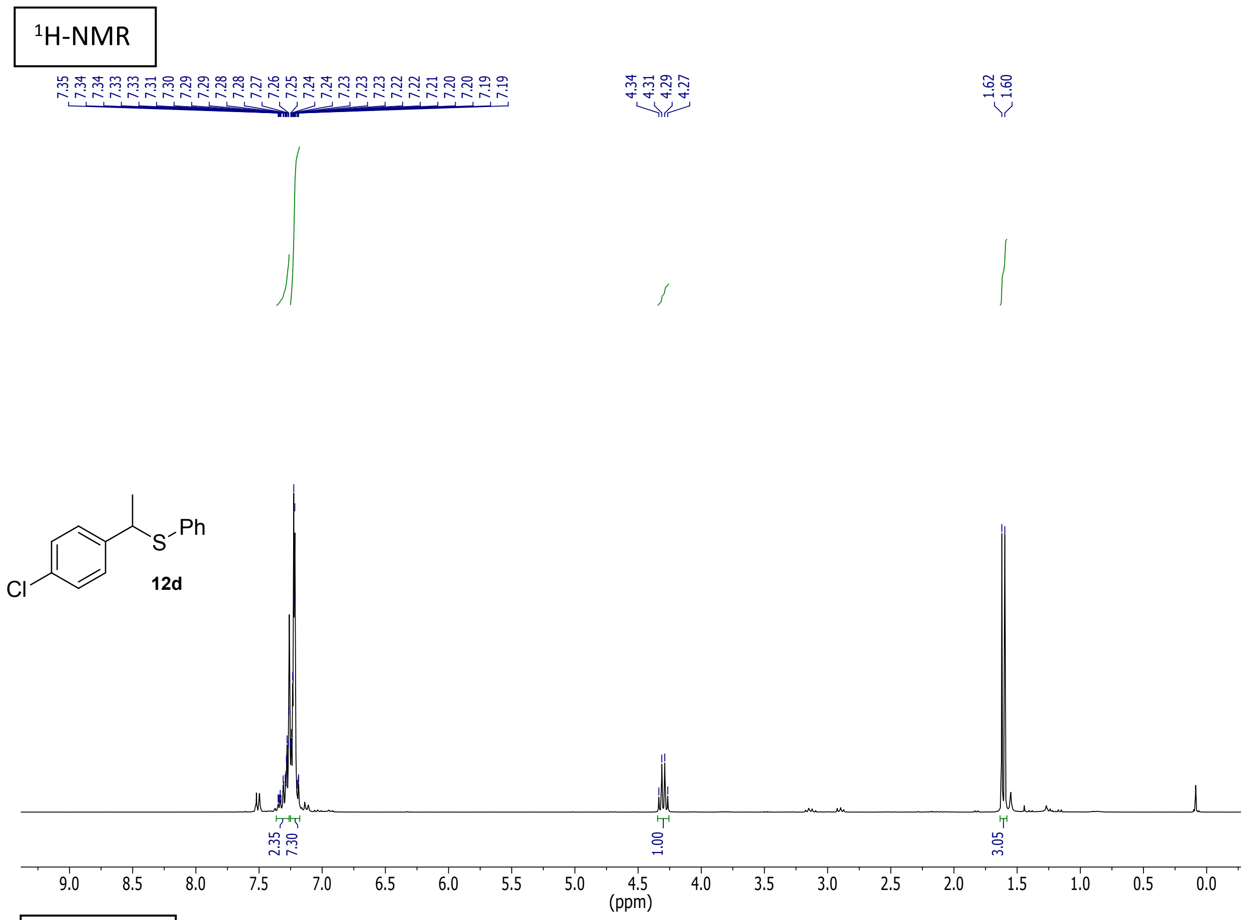

1H-NMR
7.35 7.34 7.34 7.33 7.33 7.31 7.30 7.29 7.29 7.28 7.28 7.27 7.26 7.25 7.24 7.24 7.23 7.23 7.23 7.22 7.22 7.21 7.20 7.20 7.19 7.19
4.34 4.31 4.29 4.27
1.62 1.60
Ph
S
Cl
12d
2.35 7.30
1.00
3.05
9.0 8.5 8.0 7.5 7.0 6.5 6.0 5.5 5.0 4.5 4.0 3.5 3.0 2.5 2.0 1.5 1.0 0.5 0.0
(ppm)

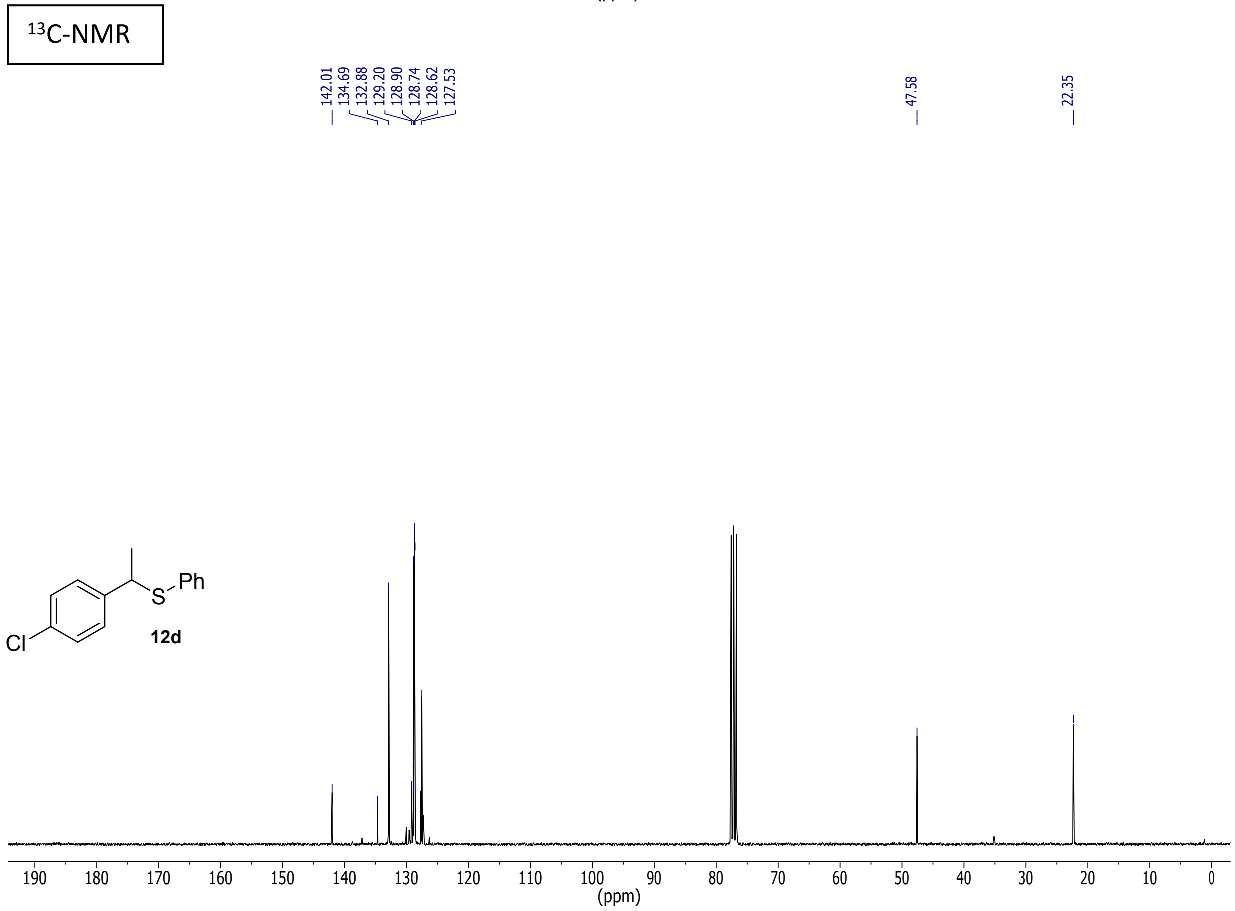

13C-NMR
142.01 134.69 132.88 129.20 128.90 128.74 128.62 127.53
47.58
22.35
Ph
S
Cl
12d
190 180 170 160 150 140 130 120 110 100 90 80 70 60 50 40 30 20 10 0
(ppm)
